# Membrane Sphingomyelin in Host Cells Is Essential for Nucleocapsid Penetration into the Cytoplasm after Hemifusion during Rubella Virus Entry

**DOI:** 10.1128/mbio.01698-22

**Published:** 2022-11-08

**Authors:** Yoshio Mori, Masafumi Sakata, Shota Sakai, Toru Okamoto, Yuichiro Nakatsu, Shuhei Taguwa, Noriyuki Otsuki, Yusuke Maeda, Kentaro Hanada, Yoshiharu Matsuura, Makoto Takeda

**Affiliations:** a Department of Virology, National Institute of Infectious Diseases, Musashimurayama, Tokyo, Japan; b Department of Biochemistry and Cell Biology, National Institute of Infectious Diseases, Shinjuku, Tokyo, Japan; c Institute for Advanced Co-Creation Studies, Osaka University, Suita, Osaka, Japan; d Center for Infectious Disease Education and Research, Osaka University, Suita, Osaka, Japan; e Laboratory of Virus Control, Research Institute for Microbial Diseases, Osaka University, Suita, Osaka, Japan; f Laboratory of Viral Dynamism Research, Research Institute for Microbial Diseases, Osaka University, Suita, Osaka, Japan; g Department of Quality Assurance, Radiation Safety, and Information System, National Institute of Infectious Diseases, Shinjuku, Tokyo, Japan; Purdue University; University of Pennsylvania

**Keywords:** rubella virus, fusion, hemifusion, sphingomyelin, CRISPR/Cas9, membrane fusion

## Abstract

The lipid composition of the host cell membrane is one of the key determinants of the entry of enveloped viruses into cells. To elucidate the detailed mechanisms behind the cell entry of rubella virus (RuV), one of the enveloped viruses, we searched for host factors involved in such entry by using CRISPR/Cas9 genome-wide knockout screening, and we found sphingomyelin synthase 1 (SMS1), encoded by the *SGMS1* gene, as a candidate. RuV growth was strictly suppressed in *SGMS1*-knockout cells and was completely recovered by the overexpression of enzymatically active SMS1 and partially recovered by that of SMS2, another member of the SMS family, but not by that of enzymatically inactive SMS1. An entry assay using pseudotyped vesicular stomatitis virus possessing RuV envelope proteins revealed that sphingomyelin generated by SMSs is crucial for at least RuV entry. In *SGMS1*-knockout cells, lipid mixing between the RuV envelope membrane and the membrane of host cells occurred, but entry of the RuV genome from the viral particles into the cytoplasm was strongly inhibited. This indicates that sphingomyelin produced by SMSs is essential for the formation of membrane pores after hemifusion occurs during RuV entry.

## INTRODUCTION

The interaction of a virus with the host cell membrane is an essential step in the entry of the virus into the cell, one of the key determinants of which is the lipid composition of the host membrane. Sphingomyelin (SM) is the most abundant sphingolipid in the membranes of eukaryotic cells and is involved, together with cholesterol, in the formation of lipid microdomains in cell membranes. Studies have reported that the cell entry of various viruses, including Ebola virus (1), pseudorabies virus (2), influenza A virus (3), and Japanese encephalitis virus (4), is inhibited by the depletion of cellular sphingomyelin. Other studies have shown that the presence of certain sphingolipids, including sphingomyelin, is essential for the fusion of liposomes and planar membranes by members of the genus *Alphavirus* (5–11). Previously, we reported that rubella virus (RuV) directly binds to SM and the cholesterol-rich membrane domain and is involved in cell entry (12). SM is intracellularly generated by sphingomyelin synthase (SMS) from ceramide and phosphatidylcholine (reviewed in reference 13). SMS has three isoforms, SMS1, SMS2, and SMS-related 1, which are encoded by the *SGMS1*, *SGMS2*, and *SMSr* genes, respectively, and only SMS1 and SMS2 have catalytic activity for SM synthesis. SMS1 is located in the Golgi apparatus, while SMS2 is located in the plasma membrane and the Golgi apparatus. SMS1 mainly controls the amount of SM in whole cells.

Rubella is caused by infection with RuV and usually presents as a mild illness characterized by low-grade fever, a short-lived morbilliform rash, and lymphadenopathy. The most serious concern with this illness is that the infection of pregnant women early during their pregnancy may result in miscarriage, stillbirth, or infants born with birth defects, known as congenital rubella syndrome (CRS). A previous study estimated that 105,000 cases of CRS occurred globally in 2010 (14). RuV is an enveloped, single-stranded, positive-sense RNA virus known as Rubivirus rubellae, within the genus *Rubivirus*, in the family *Matonaviridae*. The RuV E1 glycoprotein shares the structure and function of class II fusion proteins with the E1 proteins of *Alphavirus* and the E proteins of *Flavivirus* (15) and participates in low-pH-dependent membrane fusion at the early endosome stage (16).

In this study, to elucidate the detailed mechanism by which RuV enters cells, we used CRISPR-Cas9 library screening to search for host factors involved in this process and identified SMS1 as a candidate. Knockout of the *SGMS1* gene had little effect on the lipid mixing of the RuV envelope membrane with the cell membrane but strongly inhibited the penetration of the nucleocapsid into the cytoplasm. This inhibition was cancelled by the overexpression of SMS1 or SMS2, but not by that of enzymatically inactive SMS1, indicating that SM synthesized by SMSs is crucial for nucleocapsid penetration into the cytoplasm after hemifusion during rubella virus entry.

## RESULTS

### Genome-wide CRISPR/Cas9 screening identified SMS1 as an essential factor for the cell entry of RuV.

To identify host factors responsible for RuV entry, we applied a negative screening approach using a genome-wide knockout library in three human placenta choriocarcinoma cell lines: JAR, JEG3, and JAR4. Our previous study (17) showed that JAR and JEG3 cells are susceptible to RuV at a level comparable to that of Vero cells, an African green monkey cell line commonly used in research on this virus. The JAR4 cell line is a mitochrondrial antiviral signaling protein (MAVS)- and protein kinase R (PKR)-deficient clone generated from JAR cells by CRISPR/Cas9 gene editing (see Fig. S1A and B in the supplemental material). The maximum production of progeny RuV was increased in JAR4 cells compared with that in the original cells (Fig. S1C), and the use of recombinant RuV encoding p150-AG1, a fusion protein of green fluorescent protein AG1 and viral nonstructural protein p150 (18), allowed us to monitor viral replication by measuring fluorescence in live cells (Fig. S1D). Although RuV has low cytotoxicity for JAR and JAR4 cells, incubation with a medium containing an inhibitor against the BCL-2 family, ABT-737 or A-1331852, promoted cell death caused by RuV infection (Fig. S1E), as previously reported in studies with flaviviruses (19).

10.1128/mbio.01698-22.1 FIG S1 Establishment of the JAR4 clone cell line with *MAVS* and *PKR* gene editing. (A) The target region for the *MAVS* or *PKR* gene-specific sgRNAs in the genomic DNA of JAR4 clone. Two alleles with different indels were found in exon 5 of the *MAVS* gene and exon 3 of the *PKR* gene of the JAR4 clone (allele 1 and allele 2). (B) Protein expression of MAVS, PKR, and GAPDH in the parental JAR cells and JAR4 clone. (C) Growth kinetics of RuV in the parental JAR cells and JAR4 clone. Each cell line was inoculated with the TO-336WT-AG1 RuV strain at an MOI of 4, and the supernatants were harvested at 0, 1, 2, 3, or 4 days after incubation. The infectious titers of RuV in the supernatants are represented as means and standard deviations of triplicate samples. (D) Fluorescent microscope images of the parental JAR and JAR4 clone inoculated with the TO-336WT-AG1 strain. These cells were inoculated in the same manner as for panel C and fixed 3 days after inoculation. Expression of the p150-AG1 fusion protein was visualized by green signal. Nuclei were stained with DAPI (blue). (E) Viability of JAR4 clone line infected with the TO-336WT-AG1 strain of RuV. JAR4 cells inoculated with mock or TO-336WT-AG1 RuV strain were incubated in the presence of 1 μM A-1331852 or 1 μM ABT-737 for 5 days and then stained with crystal violet. Download FIG S1, TIF file, 1.0 MB. Copyright © 2022 Mori et al. 2022 Mori et al. https://creativecommons.org/licenses/by/4.0/ This content is distributed under the terms of the Creative Commons Attribution 4.0 International license.

**FIG 1:**
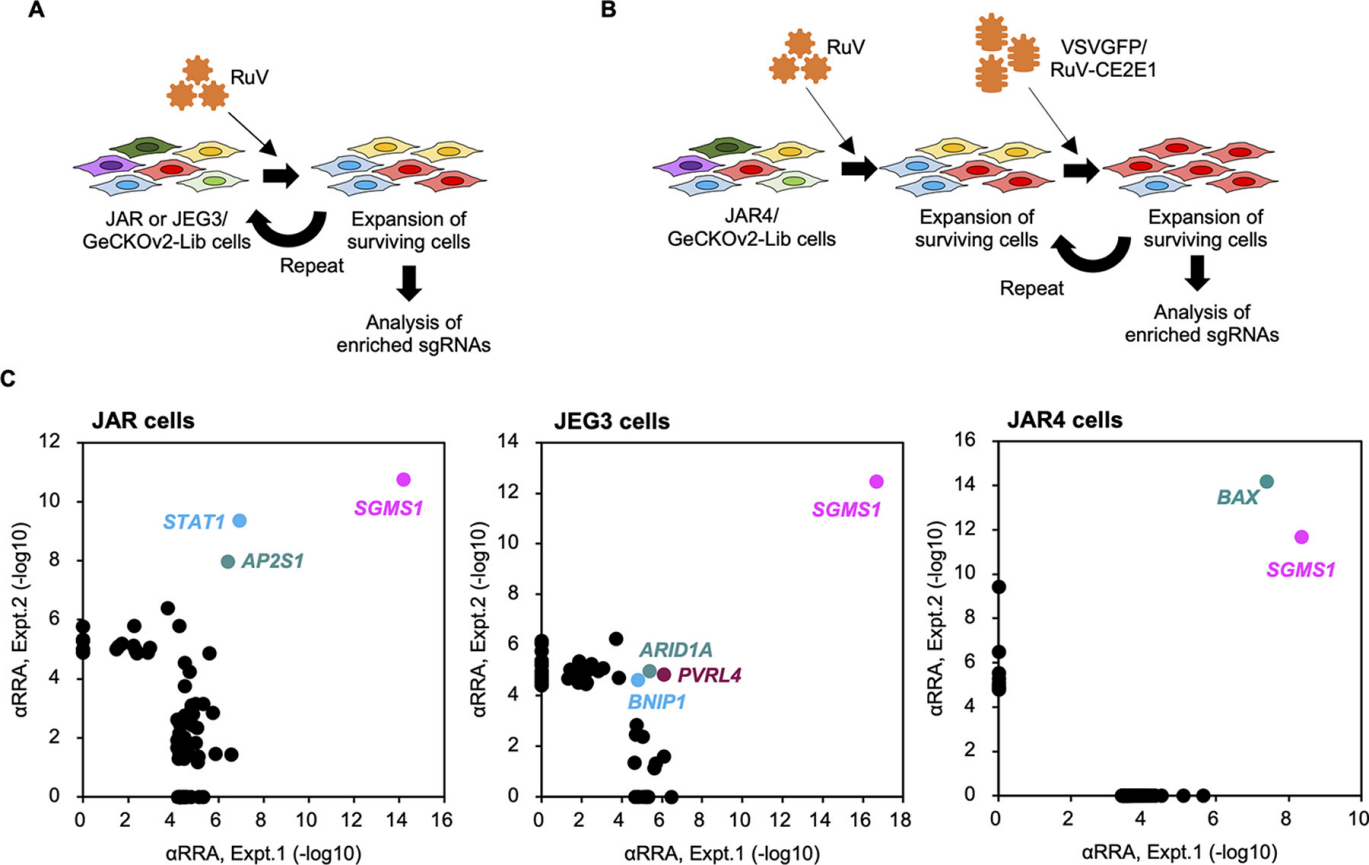
Screening of host factors essential for RuV propagation in human placenta choriocarcinoma cell lines. (A) Experimental workflow for genome-wide CRISPR/Cas9 screening in JAR or JEG3 cell line. JAR/GeCKOv2-Lib and JEG3/GeCKOv2-Lib cells were inoculated with the RuV Cendhill and HPV-77 strains, respectively, and incubated for approximately 7 days. For incubation of JAR-GeCKOv2-Lib cells, the BCL-2 inhibitor ABT-737 was added to the medium to enhance the apoptosis of infected cells. The surviving cells were passaged, inoculated with RuV again, and further incubated for 7 days. Finally, the sgRNAs enriched in surviving cells were determined. (B) Experimental workflow for genome-wide CRISPR/Cas9 screening in JAR4 cell line. JAR4/GeCKOv2-Lib cells were inoculated with the RuV TO-333Vac-AG1 strain and incubated in the presence of 1 μM A-1331852 to enhance apoptosis induced by RuV infection. Surviving cells were further inoculated with VSVGFP-RV/CE2E1 twice. Finally, sgRNAs enriched in surviving cells were determined. (C) Enrichment of sgRNAs in three series of screenings. Overall, 81, 57, and 59 genes for which sgRNAs were significantly enriched in either of the two screens using the JAR, JEG3, and JAR4 cell lines, respectively, were plotted with the adjusted robust rank aggregation method (αRRA) values. The names of genes that were significantly enriched in both experiments are indicated.

The JAR/GeCKOv2-Lib and JEG3/GeCKOv2-Lib cells were infected twice with RuV (Fig. 1A) while, to limit identification to host factors involved in RuV entry, the JAR4/GeCKOv2-Lib cells were infected once with RuV and then twice with pseudotyped vesicular stomatitis virus (VSV) possessing RuV envelope proteins (Fig. 1B). Finally, the enriched single guide RNAs (sgRNAs) were identified in the surviving cells by deep sequencing. On the basis of the analysis of sgRNAs identified in the range of 2.4 to 4.3 million reads in each cell pool, sgRNAs targeting three, four, and two genes were found to be significantly enriched in both of two independent experiments during the screening using JAR, JEG3, and JAR4 cells, respectively (Fig. 1C; Tables S1 to S3). Among them, only the *SGMS1* gene encoding SMS1 was shared by all screenings using the three cell lines. In this study, we focused on the *SGMS1* gene and further investigated the effects of SMS1 on the RuV life cycle.

10.1128/mbio.01698-22.6 TABLE S1 Statistical data for 81 genes significantly enriched in either or both of the two independent screens using the JAR-GeCKOv2-Lib cell line. Download Table S1, XLSX file, 0.02 MB. Copyright © 2022 Mori et al. 2022 Mori et al. https://creativecommons.org/licenses/by/4.0/ This content is distributed under the terms of the Creative Commons Attribution 4.0 International license.

### Enzymatically active SMSs are essential for RuV propagation.

**FIG 2:**
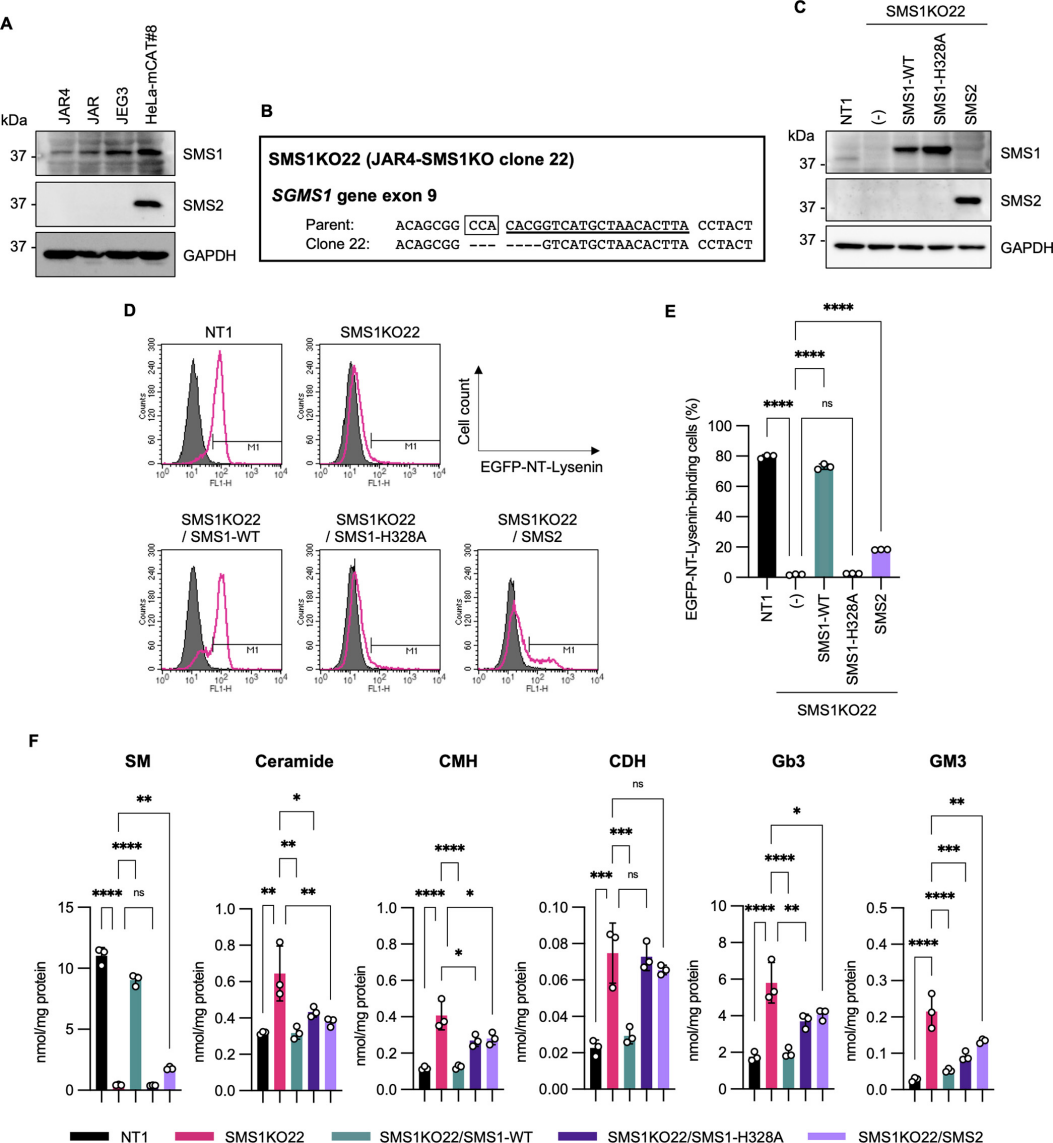
Preparation and characterization of the cell lines used in the present study. (A) Detection of SMS1, SMS2, and GAPDH in JAR, JAR4, JEG3, and HeLa-mCAT#8 cell lines by immunoblotting. (B) Nucleotide sequences around the target region for the *SGMS1* gene-specific sgRNA in exon 9 of the *SGMS1* gene of the SMS1KO22 clone (clone 22) were aligned with those of the parental JAR4 cells (parent). Target sequences for the sgRNA and following protospacer adjacent motifs are indicated by underlined and boxed sequences of the parental JAR4 cells, respectively. The SMS1KO22 clone has a homozygous 7-nucleotide deletion in the *SGMS1* gene. (C) Detection of SMS1, SMS2, and GAPDH in JAR4-derived cell lines NT1, SMS1KO22, SMS1KO22/SMS1-WT, SMS1KO22/SMS1-H328A, and SMS1KO22/SMS2 by immunoblotting. (D) Detection of clustered SM on the surface of JAR4-derived cell lines NT1, SMS1KO22, SMS1KO22/SMS1-WT, SMS1KO22/SMS1-H328A, and SMS1KO22/SMS2. Cells were treated with EGFP-NT-lysenin and analyzed by flow cytometry. Histograms with magenta line and gray fill represent EGFP-NT-lysenin-treated and untreated cells, respectively. Cells in the M1 region (fluorescent intensity of 50 and above) were defined as positive for binding to EGFP-NT-lysenin. The means and standard deviations of triplicate samples are reported in panel E. (E) Significant differences as determined by one-way analysis of variance (ANOVA) with Tukey’s *post hoc* multiple-comparison tests are indicated by asterisks: ****, *P < *0.0001; ns, not significant. (F) Quantification of sphingolipids in JAR4-derived cell lines NT1, SMS1KO22, SMS1KO22/SMS1-WT, SMS1KO22/SMS1-H328A, and SMS1KO22/SMS2 by LC-MS analysis. Lipids were extracted from cells and quantified by LC-MS. The graphs indicate the means and standard deviations of triplicate samples. Significant differences by one-way ANOVA with Tukey’s *post hoc* multiple-comparison tests are indicated by asterisks: *, *P < *0.05; **, *P < *0.01; ***, *P < *0.001; ****, *P < *0.0001; ns, not significant. CMH, ceramide monohexoside (glucosylceramide and galactosylceramide); CDH, ceramide dihexoside (lactosylceramide and galabiosylceramide); Gb3, trisaccharide globo-series sphingolipid; GM3, monosialodihexosylganglioside.

Although two isoforms within the SMS family, SMS1 and SMS2, possess sphingomyelin synthase activity, only SMS1 was detectable in JAR4, JAR, and JEG3 cells by immunoblotting (Fig. 2A). The fact that the *SGMS1* gene was found in all the screenings in the three cell lines suggests that the role of SMS1 is not affected by the knockout of *MAVS* and *PKR* genes introduced in JAR4 cells. Therefore, in subsequent experiments, JAR4 cells were used primarily because of their advantages in experiments on RuV infection. To examine the role of SMS1 in RuV propagation, an *SGMS1* gene-edited JAR4 cell clone, SMS1KO22, was prepared. This clone has a homozygous 7-nucleotide deletion in exon 9 of the *SGMS1* gene (Fig. 2B), which abolishes SMS1 expression (Fig. 2C). Enhanced green fluorescent protein-nontoxic lysenin (EGFP-NT-lysenin), a probe specific for clustered sphingomyelin (20), bound to the surface of NT1 cells, namely, JAR4 cells introduced with nontarget sgRNA, but not the surface of SMS1KO22 cells (Fig. 2D and E). On the basis of SMS1KO22 cells, we further prepared three bulk cell lines stably expressing SMS1 wild type (SMS1KO22/SMS1-WT), its enzyme activity-deficient mutant with an H-to-A change at position 328 (SMS1KO22/SMS1-H328A), or SMS2 (SMS1KO22/SMS2) (Fig. 2C). The binding of EGFP-NT-lysenin was restored in most SMS1KO22/SMS1-WT cells and in a small proportion of SMS1KO22/SMS2 cells, but not in SMS1KO22/SMS1-H328A cells (Fig. 2D and E). In addition, several clonal cells were obtained from bulk SMS1KO22 cells overexpressing SMS1-WT or SMS2 (Fig. S2A), and the functional differences between SMS1 and SMS2 were analyzed. As previously reported (21), SMS1 was localized to the Golgi apparatus and SMS2 was mainly localized to the plasma membrane (Fig. S2B), and the pattern of EGFP-NT-lysenin-binding was generally similar between each bulk and clonal cell (Fig. S2C). Liquid chromatography-mass spectrometry (LC-MS) analysis of sphingolipids showed that the amount of SM was significantly lower in SMS1KO22 cells than in NT1 cells, while the amounts of the other sphingolipids, including ceramide, ceramide monohexosides (CMH), ceramide dihexosides (CDH), globotriaosylceramide (Gb3), and ganglioside GM3, were increased (Fig. 2F; Table S4). Overexpression of SMS1-WT in SMS1KO22 cells reversed the changes in the amounts of both SM and other sphingolipids up to the same levels as in NT1 cells. Meanwhile, overexpression of SMS2 in SMS1KO22 cells resulted in a moderate increase in the amount of SM and a moderate-to-high reduction in the amounts of the other sphingolipids, except for CDH. Overexpression of SMS1-H328A did not increase the amount of SM at all, but the amounts of the other sphingolipids showed similar patterns as in the SMS2-expressing cells.

10.1128/mbio.01698-22.2 FIG S2 Characterization of clones of SMS1KO22 cells overexpressing SMS1-WT or SMS2. As described in Materials and Methods, SMS1-WT or SMS2 was stably overexpressed in SMS1KO22 cells to prepare pools of the cells (bulk). Three clones each were established from each pool of cells by limiting dilution. (A) Detection of SMS1, SMS2, and GAPDH in parental SMS1KO22, bulk, or clones of SMS1-WT- or SMS2-overexpressing cells by immunoblotting. (B) Subcellular localization of SMS1-WT and SMS2 overexpressed in SMS1KO22/SMS1-WT-clone 1 and SMS1KO22/SMS2-clone 1, respectively. SMS1 and GOLGA4 (a marker of *trans*-Golgi) or SMS2 and SLC3A2 (a marker of plasma membrane) were detected by immunofluorescent assay. Nuclei were stained with DAPI (blue). Representative images by confocal microscopy are shown. The inserts show enlarged images of the boxes indicated by the dashed lines. (C) Detection of clustered SM on the surface of SMS1KO22-derived cells, parental SMS1KO22, SMS1KO22/SMS1-WT (bulk or clones 1 to 3), or SMS1KO22/SMS2 (bulk or clones 1 to 3). Cells were treated with EGFP-NT-lysenin and analyzed by flow cytometry. Histograms with a magenta line and gray fill represent EGFP-NT-lysenin-treated and untreated cells, respectively. Percentages of cells within the M1 region are indicated. (D) Growth kinetics of RuV in SMS1KO22-derived cells, parental SMS1KO22 (−), SMS1KO22/SMS1-WT (bulk or clone 1), or SMS1KO22/SMS2 (bulk or clone 1). Each cell line was inoculated with the TO-336WT-AG1 RuV strain at an MOI of 10, and the supernatants were harvested at the indicated days after incubation. The infectious titers of RuV in the supernatants are represented as means and standard deviations of triplicate samples. (E) Infectivity of pseudotyped VSV in SMS1KO22-derived cells or parental SMS1KO22, SMS1KO22/SMS1-WT (bulk or clones 1 to 3), or SMS1KO22/SMS2 (bulk or clones 1 to 3). Each cell line was inoculated with firefly luciferase gene-encoding pseudotyped VSVs, VSVFLuc-ΔG (ΔG), VSVFLuc-RV/CE2E1 (CE2E1), or VSVFLuc-G (VSV-G). The firefly luciferase activity was measured at 24 h postinoculation. The graph indicates the means and standard deviations of three independent assays. Significant differences as determined by two-way ANOVA with Tukey’s *post hoc* multiple-comparison tests are indicated by asterisks: ****, *P < *0.0001; ns, not significant. Download FIG S2, TIF file, 1.4 MB. Copyright © 2022 Mori et al. 2022 Mori et al. https://creativecommons.org/licenses/by/4.0/ This content is distributed under the terms of the Creative Commons Attribution 4.0 International license.

10.1128/mbio.01698-22.7 TABLE S2 Statistical data for 57 genes significantly enriched in either or both of the two independent screens using the JEG3-GeCKOv2-Lib cell line. Download Table S2, XLSX file, 0.01 MB. Copyright © 2022 Mori et al. 2022 Mori et al. https://creativecommons.org/licenses/by/4.0/ This content is distributed under the terms of the Creative Commons Attribution 4.0 International license.

10.1128/mbio.01698-22.8 TABLE S3 Statistical data for 59 genes significantly enriched in either or both of the two independent screens using the JAR4-GeCKOv2-Lib cell line. Download Table S3, XLSX file, 0.01 MB. Copyright © 2022 Mori et al. 2022 Mori et al. https://creativecommons.org/licenses/by/4.0/ This content is distributed under the terms of the Creative Commons Attribution 4.0 International license.

10.1128/mbio.01698-22.9 TABLE S4 Quantitative analysis of sphingolipids by mass spectrometry. Download Table S4, XLSX file, 0.02 MB. Copyright © 2022 Mori et al. 2022 Mori et al. https://creativecommons.org/licenses/by/4.0/ This content is distributed under the terms of the Creative Commons Attribution 4.0 International license.

**FIG 3:**
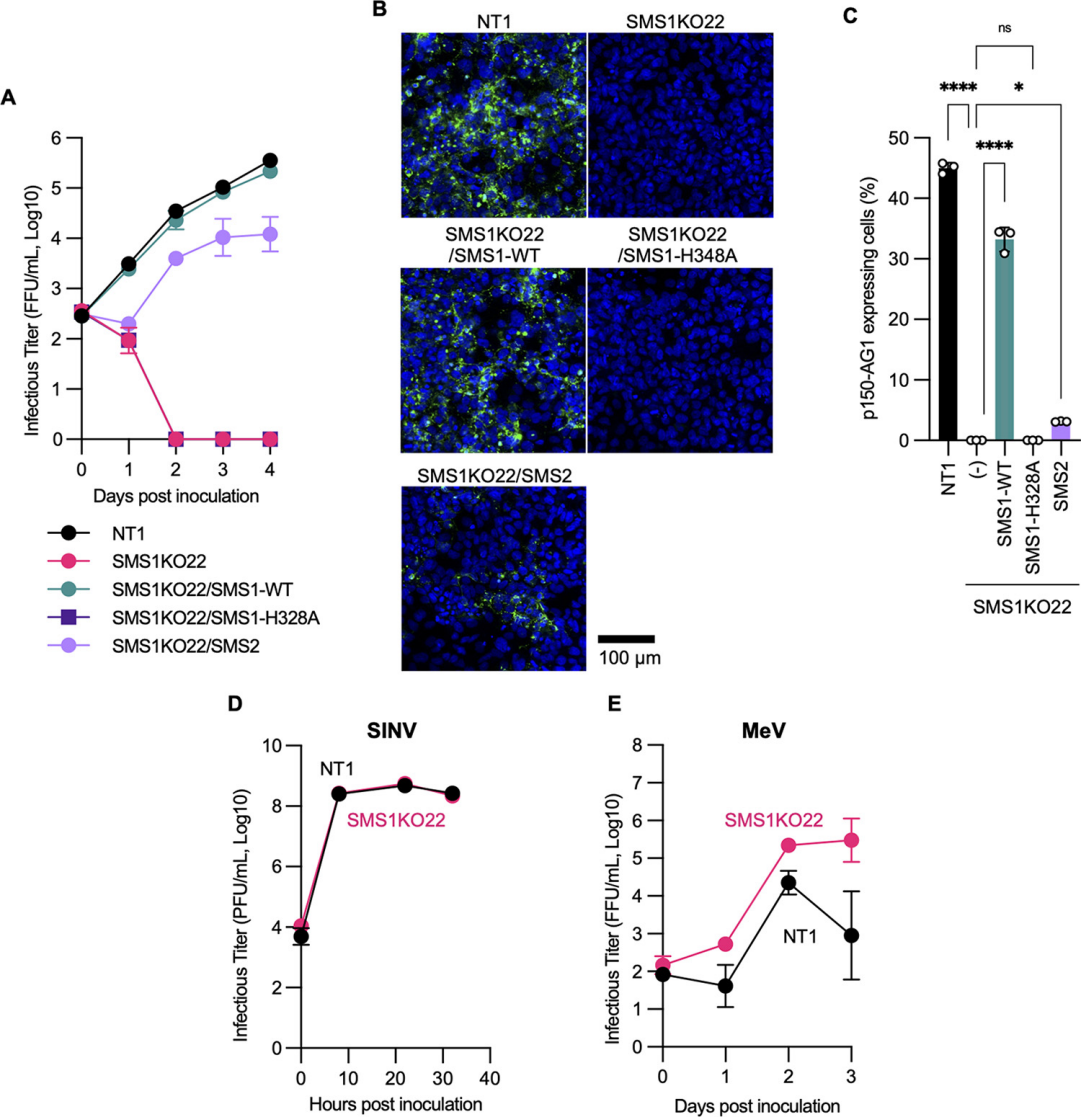
Impacts of *SGMS1* gene knockout on RuV growth. (A) Growth kinetics of RuV in JAR4-derived cell lines NT1, SMS1KO22, SMS1KO22/SMS1-WT, SMS1KO22/SMS1-H328A, and SMS1KO22/SMS2. Supernatants of each cell line inoculated with the RuV TO-336WT strain at an MOI of 10 were harvested at 0, 1, 2, 3, or 4 days after inoculation. Infectious titers in the supernatants are represented as means and standard deviations of triplicate samples. (B) Fluorescent microscopy images of each cell line, NT1, SMS1KO22, SMS1KO22/SMS1-WT, SMS1KO22/SMS1-H328A, or SMS1KO22/SMS2, inoculated with the RuV TO-336WT strain at 3 days after inoculation. Green signals indicate the expression of the p150-AG1 protein. Nuclei were stained by DAPI (blue). (C) The rate of p150-AG1-expressing cells inoculated with the RuV TO-336WT strain under the same conditions as for panel B. Cells detached with trypsin-EDTA and fixed with 4% paraformaldehyde were analyzed by flow cytometry. The graph indicates the means and standard deviations of triplicate samples. Significant differences by one-way ANOVA with Tukey’s *post hoc* multiple-comparison tests are indicated by asterisks: *, *P < *0.05; ****, *P < *0.0001; ns, not significant. (D and E) Growth kinetics of SINV (D) and MeV (E) in NT1 or SMS1KO22 cells. Infectious titers of progeny viruses are represented as means and standard deviations of triplicate samples.

**FIG 4:**
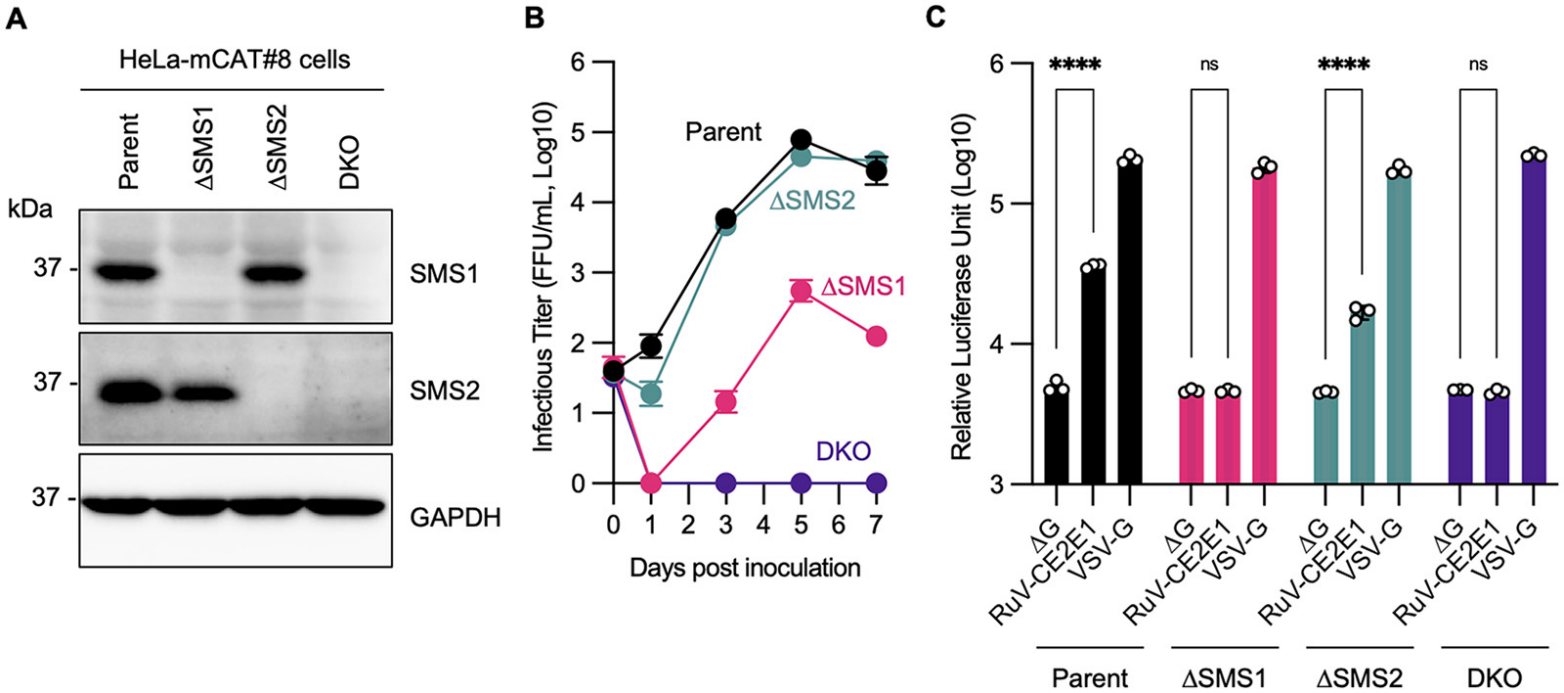
Impacts of knockout of the *SGMS1* or *SGMS2* gene on infectivity and entry of RuV in HeLa cells. (A) Detection of SMS1, SMS2, and GAPDH in HeLa-mCAT#8 cell line (Parent) and its gene-edited clones, *SGMS1* or *SGMS2* single-knockout (ΔSMS1 or ΔSMS2) and double-knockout (DKO) cells, by immunoblotting. (B) Growth kinetics of RuV in HeLa-derived cell lines. Each cell line was inoculated with the TO-336WT-AG1 RuV strain at an MOI of 10, and the supernatants were harvested at the indicated days after incubation. The infectious titers of RuV in the supernatants are represented as means and standard deviations of triplicate samples. (C) Infectivity of pseudotyped VSV in each cell line. Each cell line was inoculated with firefly luciferase gene-coding pseudotyped VSVs VSVFLuc-ΔG (ΔG), VSVFLuc-RV/CE2E1 (RuV-CE2E1), or VSVFLuc-G (VSV-G). The firefly luciferase activity was measured at 24 h postinoculation. The graph indicates the means and standard deviations of three independent assays. Significant differences as determined by two-way ANOVA with Tukey’s *post hoc* multiple-comparison tests are indicated by asterisks: ****, *P < *0.0001; ns, not significant.

When these cells were inoculated with the RuV TO-336WT-AG1 strain, it was found that the virus grew in control NT1 cells, but no virus growth was observed in SMS1KO22 cells (Fig. 3A to C). The growth of Sindbis virus (SINV), a member of the *Togaviridae* family, was comparable between NT1 and SMS1KO22 cells (Fig. 3D), and that of measles virus (MeV), a member of the *Paramyxoviridae* family, was rather enhanced in SMS1KO22 cells (Fig. 3E), suggesting that the inability to grow in SMS1KO22 cells is specific to RuV. To confirm that this inability is dependent on SMS1 expression, RuV was inoculated in a series of SMS-expressing SMS1KO22 cell lines. The results showed that RuV propagated in SMS1KO22/SMS1-WT cells to the same extent as in NT1 cells and somewhat weakly in SMS1KO22/SMS2 cells, but not in SMS1KO22-HA328A cells (Fig. 3A to C and Fig. S2D). Another cell line, human cervical epithelioid carcinoma HeLa-mCAT#8 cells, expresses both SMS1 and SMS2 endogenously, and cells with each single knockout (ΔSMS1 and ΔSMS2) and double-knockout cells (DKO) have previously been established (22) (Fig. 2A and 4A). The propagation of RuV was greatly reduced in ΔSMS1 cells, while there was little change in ΔSMS2 cells (Fig. 4B). However, no RuV propagation could be detected at all in DKO cells. Collectively, these results indicate that SMSs are essential for RuV propagation; SMS1 and SMS2 function redundantly, but SMS1, which synthesizes more SM, is thought to have a greater impact on RuV propagation.

### SMSs strictly regulate RuV entry into cells.

**FIG 5:**
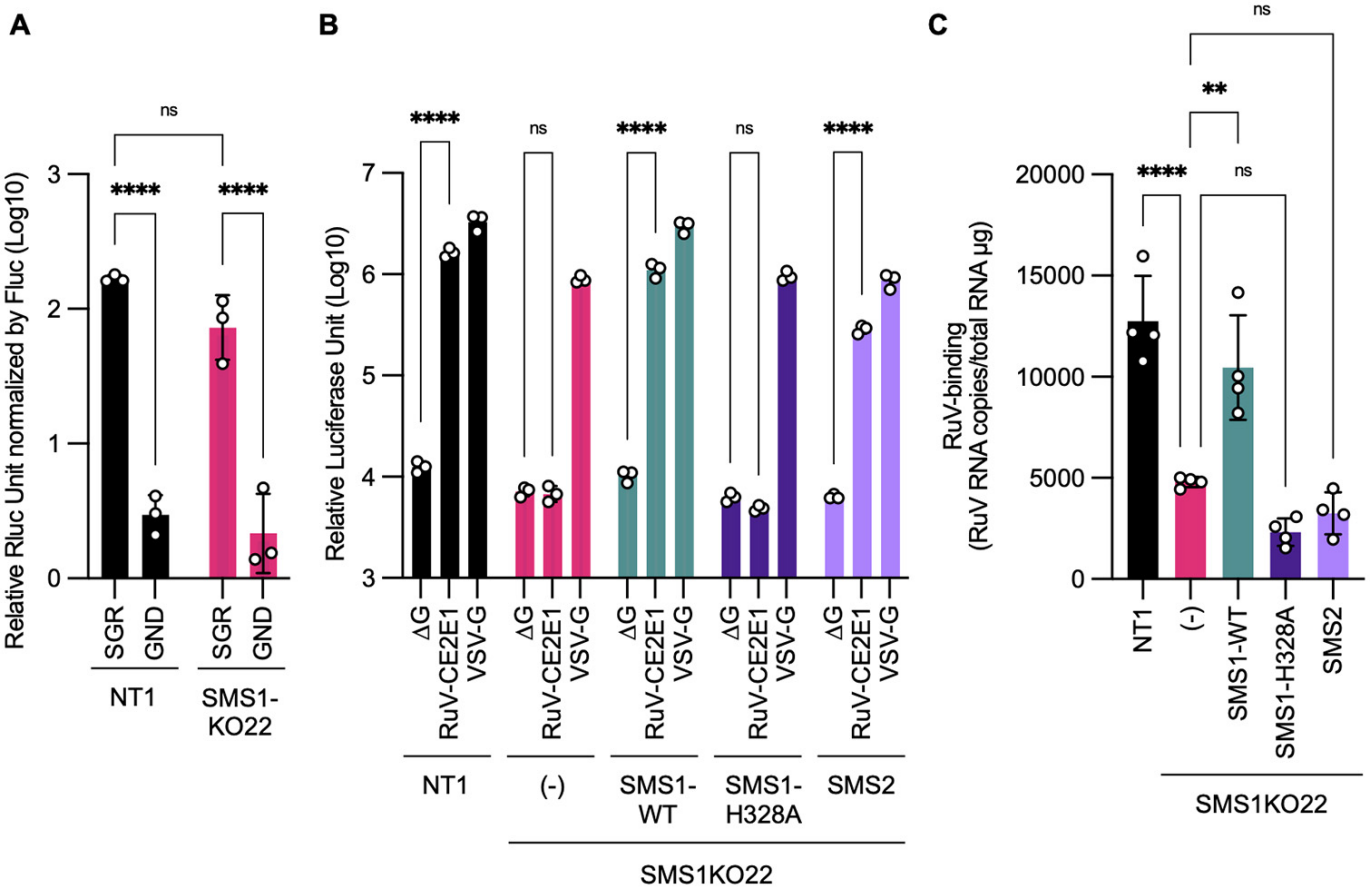
Impacts of *SGMS1* gene knockout on genome replication, entry, and binding of RuV. (A) Reporter assay of the RuV-subgenomic replicon. NT1 or SMS1KO22 cell line was transfected with the subgenomic replicon RNA HS-Rep-P2R (SGR) or replication-defective mutant HS-Rep-GND-P2R (GND), which expressed Rluc as a reporter, together with mRNAs encoding the RuV-C protein and firefly luciferase (Fluc). After 72 h of transfection, RLuc activity was determined and normalized by Fluc activity. The graph indicates the means and standard deviations of three independent assays. Significant differences as determined by two-way ANOVA with Tukey’s *post hoc* multiple-comparison tests are indicated by asterisks: ****, *P < *0.0001; ns, not significant. (B) Infectivity of pseudotyped VSVs in JAR4-derived cell lines NT1, SMS1KO22, SMS1KO22/SMS1-WT, SMS1KO22/SMS1-H328A, and SMS1KO22/SMS2. Each cell line was inoculated with Fluc gene-encoding pseudotyped VSVs, VSVFLuc-ΔG (ΔG), VSVFLuc-RV/CE2E1 (RuV-CE2E1), or VSVFLuc-G (VSV-G). The Fluc activity was measured at 24 h postinoculation. The graph indicates means and standard deviations of three independent assays. Significant differences by two-way ANOVA with Tukey’s *post hoc* multiple-comparison tests are indicated by asterisks: ****, *P < *0.0001; ns, not significant. (C) Binding of RuV to JAR4-derived cell lines NT1, SMS1KO22, SMS1KO22/SMS1-WT, SMS1KO22/SMS1-H328A, and SMS1KO22/SMS2. Each cell line was incubated with RuV at an MOI of 4 on ice for 1 h and then washed to remove unbound viruses. Total RNA was extracted from the cells, and the amount of RuV genomic RNA was determined by quantitative RT-PCR and normalized by the amount of total RNA. The graph indicates the means and standard deviations of three independent assays. Significant differences by one-way ANOVA with Tukey’s *post hoc* multiple-comparison tests are indicated by asterisks: **, *P < *0.01; ****, *P < *0.0001; ns, not significant.

To clarify at which stage of the RuV life cycle SMSs are involved, their impacts were examined separately for each step. The RuV subgenomic replicon system can evaluate the series of steps of genome replication, transcription, and translation (18). Synthetic RNA encoding a subgenomic replicon with the *Renilla luciferase* (Rluc) gene as a reporter was transfected into NT1 and SMS1KO22 cells, and no difference in RLuc expression was observed between the two cells (Fig. 5A), suggesting that SMS1 is not involved in genome replication, transcription, and translation.

The pseudotyped VSV system is available to evaluate the process by which enveloped viruses, including RuV, enter cells (17). Pseudotyped VSV possessing RuV envelope proteins, VSVFLuc/RuV-CE2E1, showed reporter expression by infection in NT1 cells, but not in SMS1KO22 cells (Fig. 5B). Infection of VSVFLuc/RuV-CE2E1 in SMS1KO22 cells was rescued by the overexpression of SMS1-WT or SMS2, but not by that of the SMS1-H328A mutant (Fig. 5B). Similar results were obtained using cell clones expressing SMS1-WT or SMS2 (Fig. S2E). Furthermore, among the panel of gene-edited HeLa/mCAT#8 cells, no infection of VSVFLuc/RuV-CE2E1 was observed in *SGMS1*-knockout cells (Fig. 4C). These results indicated that SMSs are crucial for the cell entry of RuV.

Our previous paper showed that cellular SMs are involved in the binding of RuV to cells (12). To examine the role of SMSs in RuV entry, we evaluated the binding of RuV to cells (Fig. 5C). RuV was bound to cells on ice, the unbound virus was washed out, and then the amount of virus bound to cells was measured by quantitative reverse transcription-PCR (RT-PCR). As a result, RuV binding to SMS1KO22 cells was reduced by up to about 40% of that for NT1 cells and recovered with the overexpression of SMS1-WT, but not that of SMS2 or SMS1-H328A. Because RuV binding to SMS1KO22 cells still remained and no recovery of the binding was observed with SMS2 expression, these findings suggested that reduction of the RuV-binding capacity is one of the causes of the failure of RuV propagation in SMS1KO22 cell, but this alone does not fully explain it.

### Lipid mixing between RuV and cell membrane occurs even in *SGMS1*-knockout cells.

**FIG 6:**
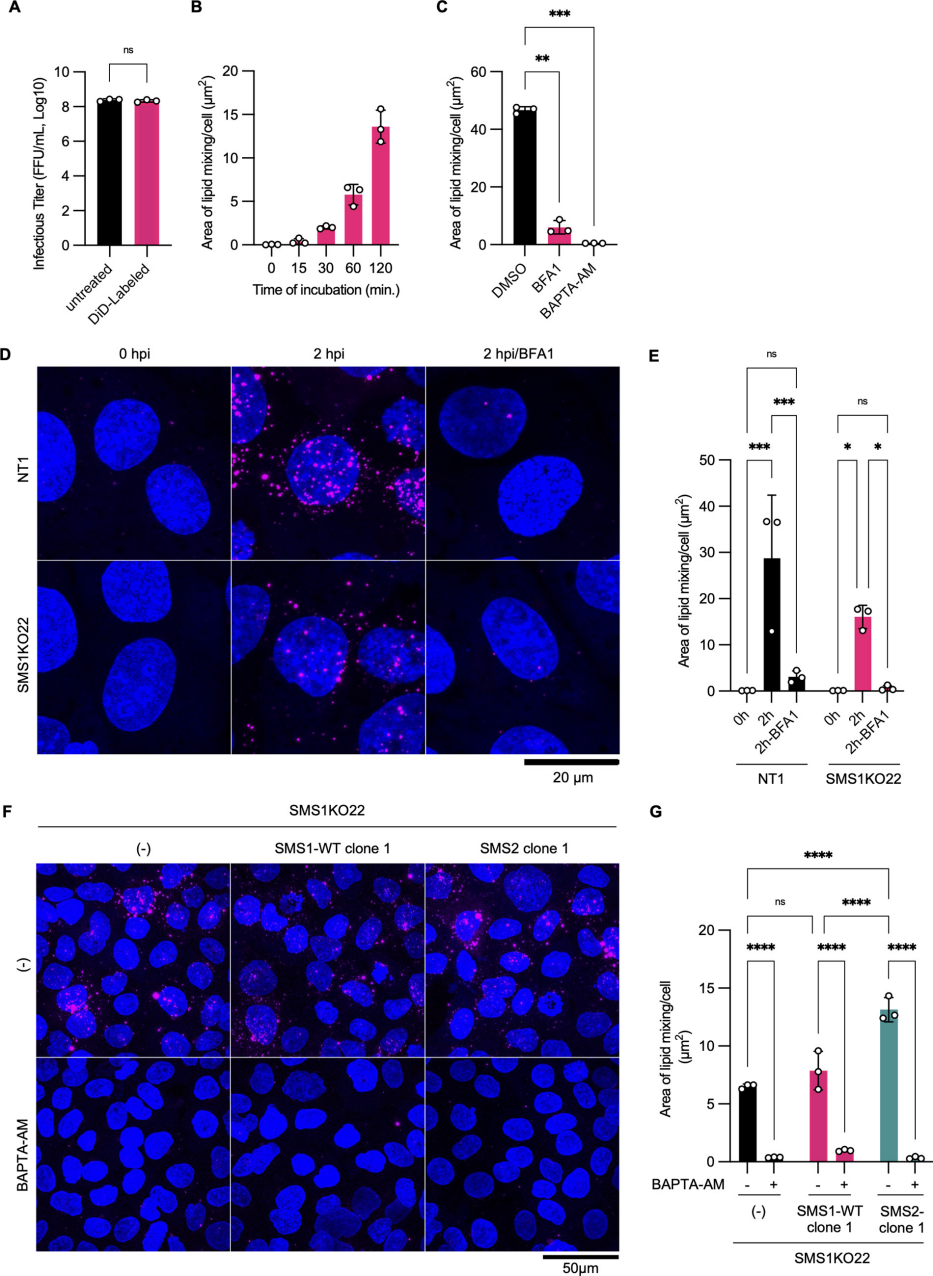
Analysis of lipid mixing between RuV envelope and cell membrane during cell entry. (A) Infectious titers of untreated or DiD-labeled RuV in NT1 cells are indicated. The significance of differences was analyzed by paired *t* test (ns, not significant). (B) After inoculation with DiD-labeled RuV at an MOI of 50 on ice, NT1 cells were incubated at 37°C for 0, 15, 30, 60, or 120 min, and areas with the DiD-fluorescent signal were determined using a confocal microscope. (C) Effects of inhibitors for RuV entry on lipid mixing. NT1 cells were incubated with a medium containing mock treatment, 100 nM Bafilomycin A1 (BFA1), or 50 μM BAPTA-AM from 1 h before to 2 h after inoculation of DiD-labeled RuV, and then areas with the DiD fluorescent signal were determined using a confocal microscope. Significant differences as determined by one-way ANOVA with Tukey’s *post hoc* multiple-comparison tests are indicated by asterisks: *, *P < *0.05. (D and F) Visualization of lipid mixing between RuV and the membrane of NT1 or SMS1KO22 cells. Cells were inoculated with DiD-labeled RuV and incubated at 37°C for 0 or 2 h (D) or 2 h (F) before fixation. For control experiments, BFA1 (final concentration, 100 nM) or BAPTA-AM (final concentration, 50 μM) was added into the medium from 1 h before RuV inoculation. Representative *z*-stack images are shown. Compartments where lipid mixing occurred were revealed by magenta signals. Nuclei were stained by Hoechst 33342 (blue). The areas of compartments where lipid mixing occurred was calculated are shown in panels E and G, respectively. (E and G) Significant differences as determined by two-way ANOVA with Tukey’s *post hoc* multiple-comparisons tests are indicated by asterisks: ****, *P < *0.0001; ***, *P < *0.001; *, *P < *0.05; ns, not significant. All graphs indicate means and standard deviations of three or four independent assays.

Enveloped viruses labeled with self-quenching concentrations of lipophilic fluorescent dye emit fluorescence when lipid mixing with the cell membrane occurs, making it possible to visualize the process of membrane fusion (16, 23–25). Labeling of a lipophilic far-red fluorescent dye, 1,1-dioctadecyl-3,3,3,3-tetramethylindodicarbocyanine (DiD), had no effect on the infectious titer of RuV (Fig. 6A). The fluorescent signals increased with time until 2 h when NT1 cells were incubated at 37°C after adsorbing the virus (Fig. 6B). Bafilomycin A1, an inhibitor of H^+^-ATPase, and BAPTA-AM, a chelator of intracellular calcium, inhibit RuV entry via endocytosis (16, 17). The expression of fluorescence was significantly inhibited by these drugs, confirming that this method can specifically detect lipid mixing by RuV (Fig. 6C to G). Using this approach, we investigated the effect of knockout of SMS1 on the lipid mixing between RuV and the cell membrane. Although the area of the DiD signal was smaller in SMS1KO22 cells than in NT1 cells, numerous punctate signals were observed intracellularly in SMS1KO22 cells after 2 h of incubation (Fig. 6D and E). ECGreen endocytosis detection reagent visualizes compartments with DsRed2-fused Rab7 (late endosomes) and partially visualizes compartments with DsRed2-fused Rab5 (early endosomes) in NT1 cells (Fig. S3A). In both NT1 and SMS1KO22 cells, DiD signals were located in the compartments stained by this reagent (Fig. S3B), indicating that lipid mixing occurred in the endosome of SMS1KO22 cells as well as in NT1 cells. Clones of SMS1KO22 cells overexpressing SMS1-WT or SMS2 had a similar or slightly larger area of lipid mixing compared with the parent cells (Fig. 6F and G), but this did not correlate well with the amount of clustered SM on the plasma membrane (Fig. S2C). Studies have reported that SMS1 deficiency affects the endocytosis, trafficking, and secretion of several proteins (26, 27), which may account for the slightly smaller lipid mixing area in SMS1KO22 cells. To rule out the effect of SMS1 deficiency on the endocytosis pathway, we examined RuV infection under conditions where this pathway was bypassed. When DiD-labeled RuV was bound to the cell surface and a low-pH medium was added, lipid mixing occurred between RuV and the plasma membrane in SMS1KO22 cells as well as NT1 cells (Fig. S4A and B). Following 48 h of incubation in the presence of ammonium chloride to inhibit endocytosis-dependent entry, infection was established in NT1 cells, but not in SMS1KO22 cells (Fig. S4A and C). The expression of SMS1-WT in SMS1KO22 cells restored the infection. These results support the idea that lipid mixing between RuV and the cell membrane occurs in SMS1KO22 cells to the same extent as in NT1 cells, but it does not lead to the establishment of infection.

10.1128/mbio.01698-22.3 FIG S3 Visualization of the endosomes by ECGreen endocytosis detection reagent. (A) NT1 cells were transfected with a plasmid, DsRed-Rab5 WT, or DsRed-Rab7 WT. One day after transfection, the cells were incubated with medium containing the ECGreen endocytosis detection reagent at 37°C for 2 h. Representative *z*-stack images of live cells observed by a confocal microscope are shown. Images in the right column are enlargements of the areas boxed with a dashed line. (B) NT1 or SMS1KO22 cells were inoculated with DiD-labeled RuV on ice for 1 h and then incubated in the presence of the ECGreen endocytosis detection reagent at 37°C for 2 h. Representative *z*-stack images of live cells observed by a confocal microscope are shown. The images of the right three columns are enlargements of the boxed areas in the left column. Outlines of cells and nuclei (N) are indicated in the images of the left column. Download FIG S3, TIF file, 2.1 MB. Copyright © 2022 Mori et al. 2022 Mori et al. https://creativecommons.org/licenses/by/4.0/ This content is distributed under the terms of the Creative Commons Attribution 4.0 International license.

10.1128/mbio.01698-22.4 FIG S4 Lipid mixing and nucleocapsid penetration during low-pH-induced entry of RuV through the plasma membrane. (A to C) NT1, SMS1KO22, or SMS1KO22/SMS1-WT cells were bound with RuV, which was labeled with the DiD lipid dye at the self-quenched concentration, followed by treatment with medium adjusted to pH 5.1 to induce lipid mixing between RuV and the plasma membrane and fixing with paraformaldehyde. One replicate was allowed to express the p150-AG1 protein (green) by incubation at 35°C for 48 h before fixation. (A) Visualization of lipid mixing between RuV and the plasma membrane and expression of the viral p150-AG1 protein. Compartments where lipid mixing occurred were revealed by magenta signals. Nuclei were stained by Hoechst 33342 (blue). Representative *z*-stack images are shown. (B) Percentages of cells with lipid mixing signals just after low-pH treatment or with no treatment (control). The graph indicates means and standard deviations of three independent assays. No significant differences as determined by two-way ANOVA with Tukey’s *post hoc* multiple-comparison tests are indicated by ns. (C) Percentages of cells expressing the p150-AG1 protein at 48 h after incubation. The graph indicates means and standard deviations of three independent assays. Significant differences as determined by one-way ANOVA with Tukey’s *post hoc* multiple-comparison tests are indicated by asterisks: **, *P < *0.01; ***, *P < *0.001. (D and E) NT1, SMS1KO22, or SMS1KO22/SMS1-WT cells were bound with RuV and then treated with medium adjusted to pH 5.1 to induce lipid mixing between RuV and the plasma membrane. After incubation at 37°C for 3 h with RPMI 1640 containing 5% FBS, cells were fixed with 4% paraformaldehyde. The RuV genome (pseudocolored in magenta) and the E1 protein (green) were stained by *in situ* hybridization and an indirect immunofluorescent assay, respectively. Nuclei were stained by DAPI (blue). (D) Representative *z*-stack images obtained by a confocal microscope are shown. The three columns on the right are enlarged images of the areas enclosed by dashed boxes in the left column. Bars for original and enlarged images indicate 20 μm and 5 μm, respectively. (E) Percentage of puncta in which RuV genomic RNA was present that colocalized with E1 protein. The graph indicates the means and standard deviations of three independent assays. Significant differences as determined by one-way ANOVA with Tukey’s *post hoc* multiple-comparison tests are indicated by an asterisk: *, *P < *0.05. Download FIG S4, TIF file, 2.7 MB. Copyright © 2022 Mori et al. 2022 Mori et al. https://creativecommons.org/licenses/by/4.0/ This content is distributed under the terms of the Creative Commons Attribution 4.0 International license.

### Penetration of RuV genomic RNA into the cytoplasm was abolished in *SGMS1*-knockout cells.

**FIG 7:**
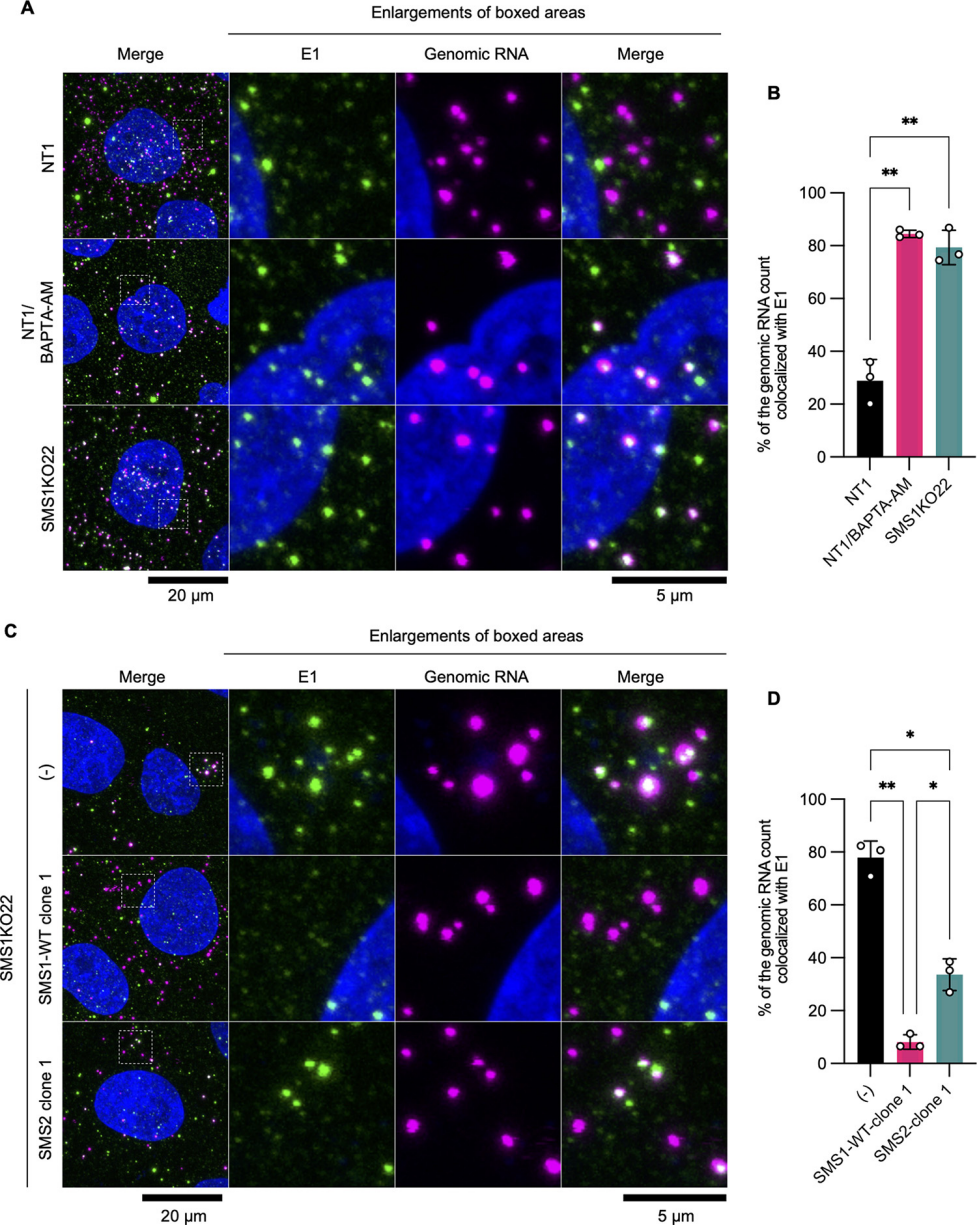
Penetration of the RuV genome into the cytoplasm. NT1 or SMS1KO22 cells (A and B), or SMS1KO22cells, SMS1KO22/SMS1-WT clone 1, or SMS1KO22/SMS2 clone 1 (C and D) were inoculated with RuV. After incubation at 37°C for 3 h, cells were fixed with 4% paraformaldehyde. For the control experiment in panel A, NT1 cells were incubated with a medium containing BAPTA-AM (final concentration, 50 μM) before inoculation of RuV at 1 h. The RuV genome (pseudocolored in magenta) and the E1 protein (green) were stained by *in situ* hybridization and indirect immunofluorescence assay, respectively. Nuclei were stained by DAPI (blue). In panels A and C, representative *z*-stack images are shown. The three columns on the right are enlarged images of the areas enclosed by dashed boxes in the left column. Bars for original and enlarged images indicate 20 μm and 5 μm, respectively. In panels B and D, percentages of puncta in which RuV genomic RNA is present and colocalized with E1 protein are indicated. The graphs indicate the means and standard deviations of three independent assays. Significant differences by one-way ANOVA with Tukey’s *post hoc* multiple-comparison tests are indicated by asterisks: **, *P < *0.01; *, *P < *0.05.

In the fusion-through-hemifusion model, it is thought that the formation and expansion of a membrane pore occur after lipid mixing between the outer leaflets of viral and cell membranes, allowing penetration of nucleocapsids into the cytoplasm (28). To examine the penetration of the RuV nucleocapsid into the cytoplasm, we evaluated the locations of the genomic RNA and E1 envelope protein at 3 h after inoculation with RuV. Because double-stranded RNA (dsRNA), an intermediate of viral genome replication, was not observed until 15 h after inoculation (Fig. S5), no *de novo*-synthesized plus-strand RNA should have been detected at the time of the experiment. When the genomic RNA is present in the viral particle, it should colocalize with the E1 protein, but once it is released from the viral particle into the cytoplasm, the RNA and E1 protein should be detected separately from each other. In NT1 cells, ~29% of the RuV genome was colocalized with the E1 protein, suggesting that the majority of the genome was released into the cytoplasm (Fig. 7A and B). In contrast, ~79% of the RuV genome remained colocalized with the E1 protein in SMS1KO22 cells. This rate was almost identical to that obtained in NT1 cells when BAPTA-AM was added at a concentration that inhibited lipid mixing. In addition, the colocalization was greatly reduced by overexpression of SMS1 and moderately by overexpression of SMS2 (Fig. 7C and D). Similar results were obtained when lipid mixing between viral and cell membranes was induced on the plasma membrane to bypass the endosomal pathway (Fig. S4D and E). Collectively, these results reveal that the failure of RuV entry in the *SGMS1*-knockout cells is mainly due to defects in the step of genomic RNA penetration into the cytoplasm following lipid mixing between RuV and the cell membrane.

10.1128/mbio.01698-22.5 FIG S5 Detection of dsRNA, an intermediate of RuV genome replication, and p150-AG1 protein. JAR4 cells were inoculated with purified RuV TO-336WT-AG1 at an MOI of 100. After incubation on ice for 1 h, the unbound viruses were removed by washing with RPMI 1640, and then the cells were incubated at 37°C for the durations indicated in the panels. The cells were fixed with 4% paraformaldehyde in PBS and then were used for immunofluorescent assay to detect dsRNA. Representative confocal microscopy images are indicated. Green, magenta (pseudocolored), and blue signals indicate p150-AG1 viral protein, dsRNA, and nucleus, respectively. Bar, 20 μm. Download FIG S5, TIF file, 2.6 MB. Copyright © 2022 Mori et al. 2022 Mori et al. https://creativecommons.org/licenses/by/4.0/ This content is distributed under the terms of the Creative Commons Attribution 4.0 International license.

## DISCUSSION

SMS1 and SMS2 are members of the SMS family, which produce SM and diacylglycerol from ceramide and phosphatidylcholine (13). Previous studies demonstrated that knockout of the genes encoding them in various types of cell results in a marked decrease in the amount of SM while simultaneously increasing the amounts of other sphingolipids (4, 22, 29). Indeed, we observed the same phenomenon with knockout of the *SGMS1* gene in JAR4 cells. Overexpression of SMS1-WT or SMS2 in SMS1KO22 cells returned the expression pattern of sphingolipids to a status comparable or intermediate to that of NT1 cells, respectively. In contrast, it was difficult to interpret the patterns of sphingolipids when enzymatically inactive SMS1-H328A was overexpressed. In this case, the SM level remained decreased, while increases in other sphingolipids were expected to occur instead. In fact, however, their levels, with the exception of CDH, were reduced compared with those in SMS1KO22 cells. This unexpected response might be accounted for by the recently elucidated mechanism that a hetero-complex of SMS1 with glucosylceramide synthase (GCS) suppresses the activity of GCS (30); if the catalytically inactive SMS1 retains the ability to inhibit GCS, its overexpression will lead it to form a complex with GCS like the active SMS1 counterpart, thereby downregulating the *de novo* synthesis of glycosphingolipids. Another unexpected response is that the increased level of ceramide in the SMS1KO22 cells was returned to the parental level by the ectopic expression of not only the SMS1-WT construct but also the SMS1-H328A construct. Although we currently have no good explanation for this response, it is conceivable that inhibiting GCS, rather than inhibiting SMS1, augments the ceramide level in the endoplasmic reticulum more, which triggers the ORMDL-dependent suppression of the serine palmitoyltransferase enzyme responsible for the initial step of *de novo* synthesis of sphingolipids (31–33). In any case, the efficiency of RuV entry correlates well with the amount of SM, but not those of the other sphingolipids, suggesting that SM synthesized by SMSs is essential for RuV entry. This would be supported by our previous study reporting that RuV entry is inhibited by SM degradation by sphingomyelin phosphodiesterase or inhibition of the SM synthetic pathway by several inhibitors (12). A limitation of this study is that there is no direct proof that SM is involved in the growth of RuV. Addition of 47 μM liposome-formed SM from egg yolk to the culture medium, as described by Hanada et al. (34), did not rescue RuV growth in SMS1KO22 cells (data not shown). However, this result was not surprising. It is known that exogenous sphingolipids are rapidly degraded in lysosomes via endocytosis to sphingosine, which serves as the material for ceramide synthesis (known as the sphingolipid salvage pathway) (reviewed in reference 35). Even if sphingosine and ceramide are supplied by this pathway, it would not lead to an increase in cellular SM in the absence of functional SMS1. Fetal bovine serum (FBS) usually contains approximately 100 μM SM (34). The fact that the amount of SM in cells is very low even under conditions where SMS1KO cells are cultured in a culture medium containing 10% FBS (i.e., containing approximately 10 μM SM) (Fig. 2F; Table S4) supports that exogenous SM does not directly increase the SM pool at the membrane of this cell line.

Because the placental choriocarcinoma cell lines used in this study (JAR/JAR4 and JEG3) did not express detectable levels of SMS2, it is thought that SMS1 within the SMS family is sufficient for RuV entry into these cells. In addition, the overexpression of SMS2 in *SGMS1*-knockout JAR4 cells restored moderate sensitivity to RuV, and knockout of the *SGMS2* gene alone had little effect on RuV growth kinetics in HeLa-mCAT#8 cells, which expressed both SMS1 and SMS2. These results indicate that SMS1 is more important than SMS2 for RuV entry. SMS1 is located in the Golgi apparatus and is thought to be involved in the bulk synthesis of SM in cells (4, 13). In contrast, SMS2, which is located in the plasma membrane, is thought to be an enzyme that converts ceramide generated on the plasma membrane to SM and has a lesser effect on the amount of SM in the entire cell (4, 36). In fact, the present study also showed that the overexpression of SMS1 in SMS1KO22 cells restored more intracellular SM and clustered SM on the plasma membrane than did the overexpression of SMS2. These different characteristics of the SMS family members may be related to the differences in their contributions to RuV entry.

**FIG 8:**
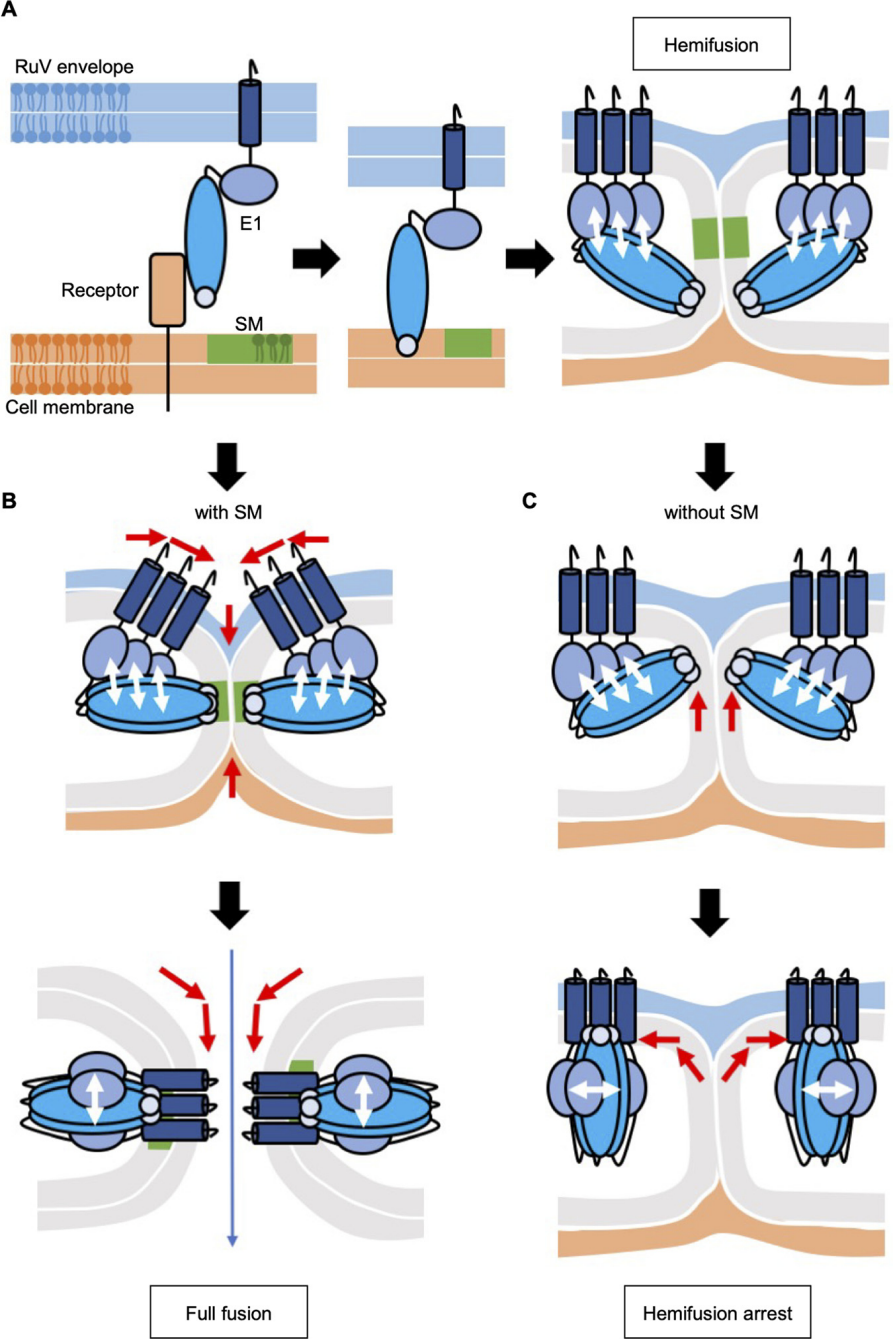
Model for cellular SM-dependent membrane fusion by RuV. (A) RuV associates with a cell by binding to cellular receptors via the E1 protein (left), and the fusion loops of the E1 protein insert into the cell membrane in a Ca^2+^-dependent manner (center). When RuV is taken up by endocytosis, exposure to a low pH causes the formation of a homotrimer of the E1 protein and a conformational change in its ectodomain. This leads to a state called hemifusion, in which only lipids between the outer leaflet of the viral envelope and that of the cell membrane are mixed (right). (B) In the presence of SM in the cell membrane, the fusion loops of the E1 protein are stabilized in the SM-rich lipid domain (green). The conformational change of the E1 protein causes lipid mixing between the inner leaflet of the viral envelope and that of the cell membrane, and membrane fusion is completed by pore formation and expansion in the membrane. Finally, the viral genome is released into the cytoplasm through the pore. (C) In the absence of SM in the cell membrane, it is thought that the anchoring by the fusion loops of the E1 protein is unstable and cannot withstand the tension caused by the conformational change of the ectodomain. This prevents lipid mixing between both inner leaflets and stops it in a dead-end hemifusion state.

How does SM synthesized by SMSs participate in the process of RuV entry? Binding to the cell surface is the first step in viral entry, and a reduction in binding was observed in SMS1KO22 cells compared with that in NT1 cells. This was consistent with our previous report describing that the depletion of SM results in reduced RuV binding at the cell surface and undetectable binding to liposomes and erythrocytes (12). However, it has been demonstrated that RuV also binds in an SM-independent manner to many cell lines that are susceptible to RuV (12). Perhaps as a result of this, RuV binding in SMS1KO22 cells remains sufficient to establish subsequent lipid mixing in endosomes, and thus the reduction of the binding is not the main cause of the inability of RuV to enter cells. Pioneering studies (37, 38) have shown that enveloped viruses fuse with host membranes in a sequential pathway called fusion-through-hemifusion (reviewed in reference 28). In this pathway, close contact of lipid bilayers of the viral envelope occurs first, followed by lipid mixing of the outer leaflet pair. At this stage, called “hemifusion,” lipid mixing of the inner leaflet pair has not occurred and the soluble contents remain separate from each other. Next, lipid mixing of the inner leaflet pair occurs, resulting in the formation of a pore between the membranes and the penetration of nucleocapsids from viral particles. Each of these steps requires energy input, and viral fusion proteins help to overcome the energy barriers by inserting their hydrophobic fusion domains (fusion peptides or fusion loops) into the target membrane and initiating conformational changes of their ectodomains triggered by receptor binding or exposure to low pH. In the *SGMS1*-knockout cells, lipid mixing between the RuV envelope and the cell membranes was established, but the subsequent penetration of the viral genome into the cytoplasm was not. This suggested that RuV failed to complete membrane fusion in *SGMS1*-knockout cells, leading to hemifusion arrest. When RuV is taken up into the cell by endocytosis, the fusion domain of the E1 protein inserts into the target membrane, and its ectodomain undergoes a conformational change and forms a homotrimer by exposure to low pH (15, 16, 25, 39). This fusion domain, consisting of two loop structures, contains a Ca^2+^-binding site and is involved in the Ca^2+^-dependent anchoring of the target membrane (15, 25). Dube et al. (16) and our present study showed that the addition of Bafilomycin A1, an inhibitor of V-type proton pump, or BAPTA-AM, a chelating agent, inhibits lipid mixing in the viral envelope and cell membranes, confirming that low-pH-induced conformational changes of E1- and Ca^2+^-dependent insertion of the fusion loops are important for the establishment of lipid mixing. Because studies have reported that RuV can bind to liposomes containing SM and cholesterol in a Ca^2+^-dependent manner (12, 16, 25), the liposome binding assay is believed to reflect the insertion of the fusion loop into the membrane. In our previous report (12), we hypothesized that the SM and cholesterol in the target membrane are important for insertion of the E1 fusion loops into the membrane because, at neutral pH, RuV binding to liposomes was not detected unless both of them were present. However, the unexpected finding in the present study, that RuV can establish lipid mixing in *SGMS1*-knockout cells, indicates that SM in the target membrane may not be essential to initiate insertion of the fusion loops into the cell membrane, but it may be involved in the stability of their membrane anchoring. Lipid rafts, which are lipid microdomains formed by SM and cholesterol, increase the local concentration of proteins and form a platform that allows them to function efficiently and stably (reviewed in reference 40). Structural studies revealed that the class II fusion proteins of flaviviruses and alphaviruses undergo conformational changes with the dynamic movement of their domains in a low-pH environment (reviewed in references 41 and 42). Their fusion loops and C-terminal transmembrane domains, which are anchored to the target and viral envelope membranes, respectively, are thought to act in membrane bending by pulling the membrane along with the conformational change of the ectodomain. Although the crystal structure of the RuV E1 protein is only available in the postfusion form, it is similar to those of other class II fusion proteins, suggesting that membrane fusion proceeds by a similar mechanism (15). In the *SGMS1*-knockout cells, the lack of SM might make the fusion loops unstable in anchoring to the membrane and unable to withstand the tension caused by a conformational change of the ectodomain, resulting in incomplete fusion (Fig. 8). In the case of influenza virus hemagglutinin (HA) proteins, replacement of the transmembrane domain with a glycophosphatidylinositol anchor (37), shortening of the transmembrane domain such that it no longer penetrates the membrane (43), or a point mutation to the N terminus of the fusion peptide (44) has been reported to cause hemifusion arrest, which may support the idea that proper anchoring to the membrane is critical for the completion of membrane fusion. Two other mechanisms could be considered. One is that, even if a membrane pore can be opened in the fusion step of RuV, its size is insufficient to release the nucleocapsid from the viral particle into the cytoplasm. A study of membrane fusion of influenza virus HA protein using a planar membrane model showed the importance of the lipid composition of the target membrane, such as sterols and sphingolipids, in membrane pore expansion (45). However, in this case, SM is rather inhibitory to membrane pore expansion. Another possibility is that SM indirectly affects the function of unidentified host proteins that act in the completion of membrane fusion by RuV. An example of how sphingolipids can affect host protein function is the case of the murine norovirus receptor CD300lf (46). Inhibition of sphingolipid synthesis does not affect the cell surface expression of CD300lf, but it does cause its conformational change, thereby inhibiting binding to murine norovirus. Based on the results of the present study, these additional possibilities cannot be ruled out, and further investigations are necessary.

This study presents a model of hemifusion arrest during cell entry by an intact virus. Previous studies showed that alphaviruses require cholesterol and sphingolipids, including SM, for membrane fusion (5, 7–11). However, those studies also indicated that lipid mixing is not induced in the absence of these lipids, suggesting that they are involved in the membrane fusion of alphaviruses in a different step than for RuV. Cell-cell membrane fusion by certain mutant HA proteins of influenza virus was shown to be interrupted at hemifusion (37, 43, 44), but hemifusion arrest was not observed upon infection with recombinant virus carrying such mutations (47). Although further studies are needed, it is expected that the present model system is very useful for analyzing the mechanism of the fusion-through-hemifusion pathway.

## MATERIALS AND METHODS

### Antibodies.

Anti-SMS1 rabbit polyclonal antibody (HPA045191; Atlas Antibodies, Bromma, Sweden), anti-SMS2 mouse monoclonal antibody (7D10; Santa Cruz Biotechnology, Dallas, TX), anti–glyceraldehyde-3-phosphate dehydrogenase (GAPDH) mouse monoclonal antibody (3H12; MBL, Nagoya, Japan), anti-RuV E1 protein mouse monoclonal antibody (2Q2070; US Biologicals, Salem, MA), anti-VSV-G protein mouse monoclonal antibody (8G5F11; Kerafast, Boston, MA), anti-MAVS rabbit monoclonal antibody (D5A9E; Cell Signaling Technology, Danvers, MA), anti-PKR rabbit monoclonal antibody (D7F7; Cell Signaling Technology), anti-p230 trans-Golgi (also known as GOLGA4) mouse monoclonal antibody (clone 15; BD Biosciences, Franklin Lakes, NJ), anti-4F2hc/CD98 (also known as SLC3A2) rabbit monoclonal antibody (Cell Signaling Technology), and anti-dsRNA mouse monoclonal antibody (J2; English and Scientific Consulting Kft., Szirak, Hungary) were used in this study. Secondary antibodies included horseradish peroxidase-conjugated goat anti-rabbit IgG (H+L) (catalog number 32460), horseradish peroxidase-conjugated goat anti-mouse IgG (H+L) (32430), Alexa Fluor 488-conjugated goat anti-mouse IgG (H+L), highly cross-adsorbed (A11029), Alexa Fluor 488-conjugated donkey anti-rabbit IgG (H+L), highly cross-adsorbed (A21206), Alexa Fluor 594-conjugated goat anti-mouse IgG (H+L), highly cross-adsorbed (A11032), Alexa Fluor 647-conjugated goat anti-mouse IgG (H+L), highly cross-adsorbed (A21235), and Alexa Fluor 647-conjugated donkey anti-rabbit IgG (H+L), highly cross-adsorbed (A31573), were purchased from Thermo Fisher Scientific (Waltham, MA).

### Plasmids.

Plasmids psPAX2 (Addgene plasmid 12260) and lentiCRISPRv2 puro (Addgene plasmid 98290) (48) were provided by Didier Trono (Ecole Polytechnique Fédérale de Lausanne, Lausanne, Switzerland) and Brett Stringer (QIMR Berghofer Medical Research Institute, Brisbane, Australia), respectively. Expression plasmids encoding fluorescent protein-fused endosome marker proteins DsRed-fused Rab5 (DsRed-Rab5 WT; Addgene plasmid 13050) (49) and DsRed-fused Rab7 (DsRed-Rab7 WT; Addgene plasmid 12661) (50) were provided by the late Richard Pagano (Mayo Clinic and Foundation, Rochester, MN). The Human GeCKOv2 CRISPR knockout pooled library (Addgene pooled library catalog numbers 1000000048 and 1000000049) (51) was provided by Feng Zhang (Broad Institute, Cambridge, MA). Plasmid pET28/His6-EGFP-NT-Lys (RIKEN BRC DNA Bank RDB13498) (20) was provided by Mitsuhiro Abe (RIKEN, Saitama, Japan).

For specific gene editing, two systems—plasmid-based and lentivirus vector-based—were used. For the editing of human *MAVS* and *PKR* genes, 20-mer guide sequences, TCAGCCCTCTGACCTCCAGC (52) and ATTCAGGACCTCCACATGAT (53), respectively, were inserted into the BsmHI site of the plasmid pSELECT-CRISPR-CAS9 (54). The resulting plasmids were designated pSELECT-CRISPR-CAS9-hMAVS and pSELECT-CRISPR-CAS9-hPKR, respectively. For lentivirus vector-based human *SGMS1* gene editing, a 20-mer guide sequence, TAAGTGTTAGCATGACCGTG, corresponding to sgRNA HGLibA43836 within the Human GeCKOv2 CRISPR knockout pooled library, was inserted into the BsmHI site of the lentiCRISPRv2 puro plasmid, in accordance with the method described by Joung et al. (55). The resulting plasmids were designated lentiCRISPRv2-hSMS1-A43836. Similarly, lentiCRISPRv2-NT1, which expressed nontarget sgRNA (guide sequence, CTGAAAAAGGAAGGAGTTGA) was prepared as described elsewhere (55).

Moloney murine leukemia virus (MMLV) packaging plasmid encoding human SMS1 protein fused with a V5 tag, porcine teschovirus-1 2A self-cleaving peptide P2A, and mCherry protein (pMMLV[EXP]-hSMS1-V5-P2A-mCherry) were prepared by VectorBuilder, Chicago, IL. To confer resistance to gene editing, two to five silent mutations were introduced into each target region for the six *SGMS1* sgRNAs (HGLibA43834 to -6 and HGLibB43781 to -3) from the Human GeCKOv2 CRISPR knockout pooled library. To prepare a plasmid encoding enzymatically inactive mutant SMS1 protein, an H328A substitution (56) was introduced into pMMLV[EXP]-hSMS1-V5-P2A-mCherry, and the resulting plasmid was designated pMMLV[EXP]-hSMS1-H328A-V5-P2A-mCherry. The plasmid pMMLV[EXP]-hSMS2-V5-P2A-mCherry was also prepared by swapping the *SGMS1* gene for the *SGMS2* gene for the expression of human SMS2 protein. Plasmids encoding subgenomic replicons of the RuV RVi/Hiroshima.JPN/0.03/[1J] strain (pHS-Rep-P2R) and its replication-defective mutant (pHS-Rep-GND-P2R) were described previously (18). Expression plasmids encoding VSV G protein (pCVSVG) and RuV C-E2-E1 proteins (pcDNA3.1-CE2E1) were reported previously (18, 57). Primers used for the preparation of plasmids are shown in Table S5.

10.1128/mbio.01698-22.10 TABLE S5 Oligonucleotides used in this study. Download Table S5, XLSX file, 0.01 MB. Copyright © 2022 Mori et al. 2022 Mori et al. https://creativecommons.org/licenses/by/4.0/ This content is distributed under the terms of the Creative Commons Attribution 4.0 International license.

### Viruses.

The RuV Cendhill and HPV-77 strains were gifts from Joseph Icenogle (Centers for Disease Control and Prevention, Atlanta, GA). The recombinant RuV TO-336-Vac and TO-336-WT strains were as reported previously (58). The recombinant TO-336-Vac and TO-336-WT strains expressing the nonstructural protein P150 fused with the green fluorescent protein humanized monomer Azami Green 1 (AG1) were generated as described previously (18). Briefly, an in-frame insertion of the *AG1* gene was introduced into the nonstructural protein *p150* gene at amino acid residues 717 and 718. These recombinant RuV strains were designated TO-336Vac-AG1 and TO-366WT-AG1. The stocks of RuV were propagated in BHK cells. The SINV M2215 strain was a kind gift from Chang-Kweng Lim. The MeV IC323/Ed-H-EGFP strain, a recombinant virus expressing EGFP and possessing the H protein derived from the Edmonston vaccine strain, was prepared as described previously (59). The firefly luciferase (Fluc) gene-coding pseudotyped VSVs, VSVFLuc-RV/CE2E1, VSVFLuc-G, and VSVFLuc-ΔG, which have envelope proteins from RuV, VSV, and mock, respectively, and the GFP gene-encoding pseudotyped VSV, possessing the RuV envelope proteins VSVGFP-RV/CE2E1, were produced as described previously (17). Although pseudotyped VSVs possessing RuV envelope proteins can be produced by expressing the RuV whole structural proteins (CE2E1) or only envelope proteins (E2E1), we used the CE2E1 construct to obtain the pseudotyped virus with higher infectious titer.

### Cells.

Human choriocarcinoma JAR (HTB-144) and JEG3 (HTB-36), human embryonic kidney 293T (CRL-3216), and African green monkey kidney Vero (CCL-81) cell lines were purchased from the American Type Culture Collection (Manassas, VA) and were maintained as described previously (17). HeLa-mCAT#8 cells and their gene-edited clones (SMS1KO, SMS2KO, and DKO) were kind gifts from Toshiyuki Yamaji (National Institute of Infectious Diseases, Tokyo, Japan) and were maintained as previously described (22). Retroviral packaging Plat-GP cells were purchased from Cell Biolabs (San Diego, CA) and were maintained in Dulbecco’s modified Eagle’s medium containing 10% FBS, 100 U/mL penicillin, 100 U/mL streptomycin, and 10 μg/mL blasticidin. To knock out *MAVS* and *PKR* genes, JAR cells were transfected with pSELECT-CRISPR-Cas9-hMAVS and pSELECT-CRISPR-Cas9-hPKR simultaneously using the Neon transfection system (Thermo Fisher Scientific). The transfected cells were selected with a medium containing 1 μg/mL puromycin from 1 to 3 days after transfection. The cells were grown in a culture medium without puromycin, and the cell clones that recovered their sensitivity to puromycin were selected. One of these clones, JAR4, was used in this study. The indels of *MAVS* and *PKR* genes in JAR4 cells were analyzed by sequencing of nucleotides around the sgRNA-targeting region.

### Human genome-scale CRISPR-Cas9 knockout library.

293T cells were transfected with pCVSVG, psPAX2, and human GeCKO v2 CRISPR knockout half-pooled library A (~65,000 sgRNA) or B (~58,000 sgRNA) by using the *Trans*IT LT1 reagent (Mirus Bio, Madison, WI). At 48 h after transfection, the culture medium was harvested and centrifuged at 10,000 × *g* for 10 min at 4°C, and the resulting supernatant was stored at −80°C until use. The titer of the lentiviral pool in the supernatant was determined by assessing the viability of the lentiviral pool-inoculated target cell lines (JAR, JAR4, and JEG3) in the presence of 1 μg/mL puromycin. One hundred million of the target cells were infected with the lentivirus pool (half-pooled A or B) at a multiplicity of infection (MOI) of approximately 0.3 and maintained in the presence of 1 μg/mL puromycin to remove uninfected cells. Theoretically, a single sgRNA was transduced in ~460 and ~520 cells per 50 million of the resulting GeCKOv2-A and GeCKOv2-B library cells, respectively.

### Genome-wide knockout screening.

Fifty million each of GeCKOv2-A and GeCKOv2-B library cells made from JAR, JEG3, and JAR4 cells were mixed to prepare the whole-library cells, designated JAR-GeCKOv2-Lib, JEG3-GeCKOv2-Lib, and JAR4-GeCKOv2-Lib cells, respectively. For screening using JAR-GeCKOv2-Lib cells and JEG3-GeCKOv2-Lib cells, the cells were inoculated with the Cendhill and HPV-77 strains, respectively, at an MOI of 10, due to their high cytotoxicity. The cells were incubated at 35°C for approximately 7 days. For incubation of JAR-GeCKOv2-Lib cells, medium containing 1 μM ABT-737 (Abcam, Cambridge, UK) was used. The surviving cells were passaged, inoculated with RuV again, and further incubated for 7 days. For screening using JAR4-GeCKOv2-Lib cells, the library cells were inoculated with the TO-336VAC-717AG1 strain at an MOI of 10. After 3 days of incubation at 35°C, A-1331852 (Selleckchem, Houston, TX) was added to the medium at a concentration of 1 μM to enhance apoptosis induced by RuV infection. The surviving cells were allowed to expand, passaged five times, and incubated for 1 month. The cells were then inoculated with VSVGFP-RV/CE2E1 at an MOI of 1 twice with a 1-week interval. The genomic DNA was extracted from 1 × 10^7^ of the original library cells (control cells) or the selected cells using a NucleoSpin tissue kit (Macherey-Nagel, Düren, Germany). These screenings were performed in duplicate. Amplicons containing sgRNA integrants from two control cells and two selected cells were prepared by 10 forward primers with different lengths of spacer nucleotides (NGS-Lib-Fwd-1 to 10) and one each of the four reverse primers with different index sequences (NGS-Lib-Rev-1 to -4), as described previously (55) (Table S5) using PrimeSTAR GXL DNA polymerase (TaKaRa Bio, Kusatsu, Japan). Purified PCR products from four different templates were measured using a QuantiFluor dsDNA system (Promega, Madison, WI), and 4 nM each was mixed and applied for deep sequencing using a MiSeq reagent kit v3 (150 cycles; Illumina, San Diego, CA) in the MiSeq system (Illumina). The resultant FASTQ files were applied to the PinAPL-Py web application (60). At least 2.4 million reads from each sample were successfully aligned and analyzed to identify sgRNA integrants enriched in the selected cells compared with the levels in the control cells. The Sidak correction method and adjusted robust rank aggregation method (αRRA) (61) were used for the ranking of enriched sgRNAs and genes, respectively. Enrichment was considered to be significant when the *P* value was <0.01 for each test.

### Establishment of an *SGMS1*-knockout clone of JAR4 cells.

293T cells were transfected with pCVSVG, psPAX2, and lentiCRISPRv2-NT1 or lentiCRISPRv2-hSMS1-A43836, and then lentivirus CRISPR-NT1 or lentivirus CRISPR-hSMS1 was obtained. JAR4 cells were inoculated with either of the lentiviruses and incubated with a medium containing 1 μg/mL puromycin. Bulk cells obtained by inoculation with lentivirus CRISPR-NT1 were used as knockout-control NT1 cells. The clone line SMS1KO22 was obtained from JAR4 cells inoculated with lentivirus CRISPR-hSMS1. The indel of the *hSGMS1* gene was analyzed by sequencing nucleotides around the sgRNA-targeting region of the genome.

Plat-GP cells were transfected with pCVSVG and pMMLV[EXP]-hSMS1-V5-P2A-mCherry, pMMLV[EXP]-hSMS1-H328A-V5-P2A-mCherry, or pMMLV[EXP]-hSMS2-V5-P2A-mCherry, and MMLVs expressing the V5 epitope-tagged human SMS1 (MMLV-SMS1-WT), its sphingomyelin synthase activity-deficient mutant (MMLV-SMS1-H328A), and the V5 epitope-tagged human SMS2 (MMLV-SMS2) were obtained, respectively. SMS1KO22 cells were inoculated with MMLV-SMS1-WT, MMLV-SMS1-H328A, and MMLV-SMS2, and stably expressing SMS1KO22/SMS1-WT, SMS1KO22/SMS1-H328A, and SMS1KO22/SMS2 cells, respectively, were obtained.

### Immunoblotting.

Cells were washed with phosphate-buffered saline (PBS) and then lysed by sonication in Pierce radioimmunoprecipitation assay (RIPA) buffer (Thermo Fisher Scientific) containing cOmplete protease inhibitor cocktail (Sigma-Aldrich, St. Louis, MO). Aliquots of the cell lysates were mixed with sodium dodecyl sulfate (SDS) sample buffer for 5 min on ice. Polypeptides in the samples were separated by SDS-polyacrylamide gel electrophoresis (PAGE) using 5% to 20% gradient SDS-PAGE gels (Fujifilm Wako Pure Chemical, Osaka, Japan) and electroblotted onto polyvinylidene difluoride membranes (Millipore, Bedford, MA). After blocking with SuperBlock (Tris-buffered saline) blocking buffer (Thermo Fisher Scientific), the membranes were incubated overnight at 4°C with primary antibodies diluted in Can Get Signal immunoreaction enhancer solution 1 (Toyobo, Osaka, Japan). The membranes were then washed three times with PBS containing 0.05% Tween 20 (PBS-T) and incubated with horseradish peroxidase-conjugated secondary antibody diluted in Can Get Signal immunoreaction enhancer solution 2 (Toyobo) for 2 h at room temperature. After washing, the membranes were treated with SuperSignal West Femto maximum sensitivity substrate (Thermo Fisher Scientific), and the chemiluminescent signals on the membranes were detected using a ChemiDoc Touch imaging system (Bio-Rad Laboratories, Hercules, CA).

### SM detection on the cell surface by EGFP-NT-lysenin.

EGFP-tagged nontoxic-type earthworm toxin lysenin (EGFP-NT-lysenin) was prepared as described previously (20) with some modifications. Briefly, Escherichia coli BL21(DE3) pLysS strain transformed with pET28-His6-EGFP-NT-Lys was induced to express EGFP-NT-lysenin by adding 1 mM isopropyl-β-d-thiogalactopyranoside for 4 h at 37°C. The cells were lysed with an X-tractor buffer kit (TaKaRa Bio). The lysate was applied to a column filled with TALON metal affinity resin (TaKaRa Bio). After washing the resin, EGFP-NT-lysenin was eluted with elution buffer (50 mM sodium phosphate, 300 mM sodium chloride, and 150 mM imidazole) and was concentrated in PBS by ultrafiltration using Amicon Ultra-4 10K centrifugal filter devices (Merck Millipore, Burlington, MA). NT1, SMS1KO22, SMS1KO22/SMS1-WT, SMS1KO22/SMS1-H328A, or SMS1KO22/SMS2 cells were detached using trypsin-EDTA (0.05%; Thermo Fisher Scientific) and reacted with 20 μg/mL EGFP-NT-lysenin for 30 min at 4°C. Untreated cells were used as controls. After washing with PBS, the cells were analyzed using a BD FACSCalibur flow cytometer (Becton Dickinson, Franklin Lakes, NJ).

### LC-MS analysis on sphingolipids.

Cells were seeded in a 10-cm dish at a density of 2 × 10^5^ cells/dish in 10 mL of culture medium. After 48 h, cells were washed three times with PBS and harvested by detaching with a strong flow of PBS by pipetting. Some of the recovered cells were lysed in Pierce RIPA buffer, and the amount of total protein was determined with a Pierce bicinchoninic acid protein assay kit (Thermo Fisher Scientific). Then, 1 nmol of each of the internal standards, C17:0 SM, C17:0 ceramide, C17:0 lactosylceramide, C17:0 glucosylceramide, C17:0 Gb3, and d18:1-d5-C18:0 GM3 (Avanti Polar Lipids, Alabaster, AL), was added for quantification by LC-tandem MS (MS/MS). Lipids were extracted from the cells and the extract was saponified with 0.4 M KOH in methanol at 37°C for 2 h to remove glycerolipids. The alkali-stable fraction was recovered with chloroform and then washed with water. The chloroform phase was evaporated under a stream of nitrogen and redissolved in 150 μL of acetonitrile-methanol (50/50 [vol/vol]). Sphingolipids were analyzed by an LC-MS/MS system that consisted of a Prominence UFLC system (Shimadzu, Kyoto, Japan) coupled to a 3200 QTRAP system (SCIEX), as described previously (62). Briefly, extracted lipids were injected onto an InertSustain column (5-μm particle size, 2.1 (internal diameter) by 150 mm; GL Science, Tokyo, Japan). Mobile phase A was acetonitrile-methanol-formic acid (95:5:0.2 [vol/vol/vol]) containing 5 mM ammonium formate, and mobile phase B was methanol-formic acid (100:0.2 [vol/vol]) containing 5 mM ammonium formate. A sample was eluted at 0.2 mL/min through a 45-min gradient as follows: mobile phase B, 0 to 5 min at 0%, 5 to 10 min from 0% to 20%, 10 to 12 min hold at 20%, 12 to 15 min from 20% to 50%, 15 to 22 min hold at 50%, 22 to 27 min from 50% to 80%, 27 to 30 min hold, 30 to 35 min from 80% to 0%, and 35 to 45 min hold to 0%. The optimal conditions for the ionization and fragmentation of each lipid were determined. MS analysis was run in the positive ion mode with the following instrument parameters: curtain gas of 10 lb/in^2^, ion spray voltage of 5,500 V, temperature of 300°C, nebulizer gas of 30 lb/in^2^, auxiliary gas of 50 lb/in^2^, and collision cell exit potential of 10 V. The levels of declustering potential, entrance potential, and collision energy were optimized for each target. Multiple-reaction monitoring (MRM) mode was used to measure each lipid molecular species that contained different acyl chains. Each ion pair of molecular species in the MRM was monitored for 20 ms with a resolution of a unit. The individual lipid contents were calculated by relating the peak area of analyte to the peak area of the corresponding internal standard. Data acquisition and analysis were performed using Analyst software version 1.6 (SCIEX).

### Growth kinetics of viruses.

Monolayers of cells were inoculated with RuV TO-336WT-AG1 strain at an MOI of 10. Supernatants were collected from cells incubated at 35°C for the times indicated in the figures. To determine infectious titers of RuV, Vero cells were inoculated with a serial dilution of the supernatants and incubated in a medium containing 0.5% agarose for 3 days at 35°C. Foci of AG1-fluorescent signals were counted using a fluorescence microscope (BZ-X810; Keyence, Osaka, Japan), and the infectious titers were expressed as focus-forming units (FFU). Infectious titers under the detection limit of 3.3 FFU/mL were recorded as 1 FFU/mL. Monolayers of cells were inoculated with the SINV M2215 strain at an MOI of 4. The supernatants were harvested at 0, 8, 22, or 32 h after inoculation at 37°C, and the infectious titers were determined by a plaque-forming assay using BHK cells, as described previously (63, 64). NT1 or SMS1KO22 cells were inoculated with the MeV IC323/Ed-H-EGFP strain at an MOI of 0.1. Cells were harvested using cell scrapers at 0, 1, 2, or 3 days after inoculation at 37°C, and the samples were prepared by freeze-thaw and centrifugation. The infectious titers were determined by a focus-forming assay using Vero cells. Foci of EGFP-fluorescent signals were counted using a fluorescence microscope (BZ-X810).

### Infectivity analysis of RuV.

Monolayers of cells were inoculated with RuV TO-336WT-AG1 strain at an MOI of 10. After incubation at 35°C for 3 days, the cells were analyzed by fluorescence microscopy or flow cytometry. For fluorescence microscopy, the cells were fixed with 4% paraformaldehyde, treated with 0.5% Triton X-100, and then stained with 4′,6-diamidino-2-phenylindole dihydrochloride (DAPI). The cells were observed using a confocal microscope (Fluoview FV3000; Olympus, Tokyo, Japan) or a fluorescence microscope (BZ-X810). For flow cytometry, the cells were detached with trypsin-EDTA, washed with PBS containing 10% FBS, and fixed with 4% paraformaldehyde. The cells were analyzed using a flow cytometer (BD FACSCalibur). Under the cutoff condition that >99.5% of uninfected control cells were removed, the percentage of cells expressing the p150-AG1 protein was determined.

### Subgenomic replicon assay.

A subgenomic replicon assay for analyzing the RuV genome RNA replication activity was performed as described previously (12, 18) with some modifications. Briefly, the subgenomic replicon RNA HS-Rep-P2R and replication-defective HS-Rep-GND-P2R were synthesized from the plasmids pHS-Rep-P2R and pHS-Rep-GND-P2R, respectively, by *in vitro* RNA transcription using the mMESSAGE mMACHINE SP6 transcription kit (Thermo Fisher Scientific). The mRNAs encoding RuV C protein and Fluc were synthesized from the plasmid carrying each gene by *in vitro* RNA transcription using the mMESSAGE mMACHINE T7 transcription kit (Thermo Fisher Scientific). JAR4-derived cells were transfected with the replicon RNA HS-Rep-P2R or HS-Rep-GND-P2R, together with the C protein and Fluc mRNAs, using the Lipofectamine MessengerMAX transfection reagent (Thermo Fisher Scientific) and were lysed after 72 h of incubation at 35°C. The activities of Rluc and Fluc in the lysates were measured with the *Renilla* luciferase assay system (Promega) and ONE-Glo luciferase assay system (Promega), respectively, using the GloMax Explorer system (Promega). The RuV replication activity is reported as the RLuc assay value normalized to the Fluc assay value.

### Entry assay using pseudotyped VSVs.

An entry assay using pseudotyped VSVs was performed as described previously (17). Briefly, JAR4-derived cells were inoculated with Fluc gene-carrying a pseudotyped VSV (VSVFLuc-RV/CE2E1, VSVFLuc-G, or VSVFLuc-ΔG) in 96-well clear-bottomed white plates (Corning, NY). Before inoculation, VSVFLuc-RV/CE2E1 and VSVFLuc-ΔG were treated with anti-VSV-G monoclonal antibody 8G5F11 for 90 min at 4°C to neutralize carryover of VSV-G. At 24 h postinoculation, the Fluc activity was measured by the Bright-Glo assay system (Promega) and GloMAX Explorer system.

### Binding assay of RuV.

Monolayers of cells in a six-well plate were inoculated with RuV TO-336WT-AG1 strain at an MOI of 4. After incubation on ice for 1 h, cells were washed with RPMI 1640 three times, and the total RNA of the cells was extracted with TRIzol reagent (Thermo Fisher Scientific). The amounts of RuV RNA in the extracts were calculated by quantitative RT-PCR (65) and normalized by the amounts of total RNA measured with QuantiFlour RNA system (Promega). The primers and probe used in the quantitative RT-PCR are shown in Table S5.

### Fluorescence labeling of RuV particles.

An 800-mL stack of the RuV TO-336WT-AG1 strain was mixed with 200 mL of PEG-it virus precipitation solution (5×; System Biosciences, Palo Alto, CA) and incubated at 4°C overnight. The mixture was centrifuged at 1,500 × *g* for 30 min at 4°C twice. The resulting precipitation was resuspended with PBS containing 65 mM ethylenediaminetetraacetic acid (PBS-EDTA). The suspension (9 mL) was overlaid on 15% sucrose in PBS (2 mL) and then centrifuged at 49,000 × *g* for 3 h at 4°C. The precipitation was resuspended in PBS-EDTA again, overlaid on a 10% to 50% five-step discontinuous gradient of OptiPrep (Abbott Diagnostics Technologies AS, Oslo, Norway), and then centrifuged at 175,000 × *g* for 3 h at 4°C. The visible band between 20% and 30% OptiPrep layers was collected as purified RuV. Three hundred microliters of the purified RuV (1 × 10^8^ FFU) was supplemented with 7.5 μL of 100 μM Vybrant DiD cell-labeling solution (Thermo Fisher Scientific) with mixing by a vortex mixer and incubated at room temperature for 1 h. Under these conditions, the DiD dye incorporated into the lipid envelope of RuV was self-quenched. Just before use, the DiD-labeled RuV was diluted with RPMI 1640 containing 10% FBS to a concentration of 2 × 10^7^ FFU/100 μL.

### Detection of lipid mixing during cell entry of RuV.

Monolayers of NT1 or SMS1KO22 cells in eight-well chamber slides (ibiTreat-μ-Slide 8 well; ibidi, Grafelfing, Germany) were inoculated with the DiD-labeled RuV at an MOI of 50. After incubation on ice for 1 h, the unbound viruses were removed by washing with RPMI 1640 twice, and then the cells were incubated at 37°C for 0 or 2 h. For control of the membrane fusion between RuV and the endosome, cells were supplemented with 100 nM Bafilomycin A1 (EMD Chemicals, San Diego, CA) or 50 μM BAPTA-AM (Dojindo, Kumamoto, Japan) from 1 h before RuV inoculation to the end of incubation. The cells were fixed with 4% paraformaldehyde in PBS, and the nuclei were stained with 1 μg/mL Hoechst 33342 (Dojindo). The cells were observed using a confocal microscope (Fluoview FV3000). To quantify the lipid mixing, areas with the DiD fluorescent signal in *z*-stack images of approximately 150 cells from five random fields were measured using the Fiji package of ImageJ (66).

### Induction of RuV entry at the plasma membrane.

Monolayers of JAR4-derived cells in an eight-well chamber slide were inoculated with DiD-labeled RuV at an MOI of 50. After incubation on ice for 1 h, the unbound viruses were removed by washing with RPMI 1640 twice, and the cells were incubated with a low-pH (pH 5.1) culture medium or normal medium at 37°C for 15 min. For one set of the slides, the cells were fixed with 4% paraformaldehyde in PBS at this point. For another set of the slides, the cells were incubated at 35°C for 48 h with RPMI 1640 containing 10% FBS and 20 mM ammonium chloride, to prevent multiple infections and then fixed with 4% paraformaldehyde in PBS. After staining the nuclei with 1 μg/mL Hoechst 33342, the cells were observed using a confocal microscope (Fluoview FV3000) or a fluorescence microscope (BZ-X810). The cells exhibiting lipid mixing or infection were counted among over 800 cells from five random fields.

### Detection of penetration of the RuV genome into the cytoplasm.

Monolayers of JAR4-derived cells in eight-well chamber slides were inoculated with purified RuV at an MOI of 100 and incubated on ice for 1 h, after which the unbound viruses were removed by washing with RPMI 1640 twice. To detect penetration of the RuV genome into the cytoplasm via the endocytosis pathway, the cells were incubated at 37°C for 3 h. For control of membrane fusion between RuV and the endosome, cells were supplemented with 50 μM BAPTA-AM from 1 h before RuV inoculation to the end of incubation. To detect the penetration of the RuV genome into the cytoplasm after the induction of membrane fusion at the plasma membrane, the cells were incubated with a low-pH (pH 5.1) culture medium at 37°C for 15 min and then incubated at 37°C for 2 h in RPMI 1640 containing 5% FBS. These cells were fixed with 4% paraformaldehyde in PBS at room temperature for 30 min and then washed with PBS three times. The RuV genomic RNA was detected by *in situ* hybridization with a QuantiGene ViewRNA ISH cell assay kit (Thermo Fisher Scientific). The probe set was designed to detect nucleotide positions 6086 to 7266 of the positive-strand RNA of the RuV TO-336 strain (GenBank accession number AB588192). After this procedure, the cells were reacted with anti-RuV E1 mouse monoclonal antibody at a concentration of 1 μg/mL at 4°C overnight. After washing with PBS-T three times, cells were incubated with Alexa Fluor 488-conjugated goat anti-mouse IgG (H+L) antibody and DAPI at room temperature for 2 h. After washing, cells were observed using a confocal microscope (Fluoview FV3000). To ensure that empty RuV particles without the genomic RNA were not included in the calculation, the number of puncta with genomic RNA was used as the denominator and the number of colocalized E1 puncta was used as the numerator. Over 400 puncta with the genomic RNA were analyzed using the Fiji package of ImageJ.

### Cell viability assay.

JAR4 cells were inoculated with mock or RuV TO-336WT-AG1 strain at an MOI of 5 and incubated in a medium in the presence or absence of 1 μM A-1331852 or 1 μM ABT-737. At 5 days after inoculation, cells were stained with PBS containing 0.1% crystal violet and 4% formalin.

### Immunofluorescence assay for overexpressed SMS1 and SMS2 in SMS1KO22/SMS1-WT and SMS1KO22/SMS2 cells, respectively.

Cells in eight-well chamber slides were fixed with 4% paraformaldehyde and then permeabilized with 0.5% Triton X-100. SMS1KO22/SMS1-WT clone 1 was incubated with anti-SMS1 rabbit polyclonal and anti-GOLGA4 mouse monoclonal antibodies at 4°C overnight. After washing with PBS-T three times, the clone was incubated with Alexa Fluor 488-conjugated goat anti-mouse IgG (H+L) antibody, Alexa Fluor 647-conjugated donkey anti-rabbit IgG (H+L) antibody, and DAPI at room temperature for 2 h. SMS1KO22/SMS2 clone 1 was incubated with anti-SMS2 mouse monoclonal and anti-SLC3A2 rabbit monoclonal antibodies at 4°C overnight. After washing with PBS-T three times, the clone was incubated with Alexa Fluor 488-conjugated donkey anti-rabbit IgG (H+L) antibody, Alexa Fluor 647-conjugated goat anti-mouse IgG (H+L) antibody, and DAPI at room temperature for 2 h. After washing, cells were observed using a confocal microscope (Fluoview FV3000).

### Immunofluorescence assay for detection of dsRNA, an intermediate of RuV genome replication.

NT1 cells in eight-well chamber slides were inoculated with purified RuV TO-336WT-AG1 strain at an MOI of 100 and incubated on ice for 1 h. The unbound viruses were removed by washing with RPMI 1640 twice. After incubation at 37°C for 0, 1, 3, 6, 15, or 24 h, cells were fixed with 4% paraformaldehyde and then permeabilized with 0.5% Triton X-100. Cells were incubated with anti-dsRNA mouse monoclonal antibody at 4°C overnight. After washing with PBS-T three times, cells were incubated with Alexa Fluor 594-conjugated goat anti-mouse IgG (H+L) antibody and DAPI at room temperature for 2 h. After washing, cells were observed using a confocal microscope (Fluoview FV3000).

### Imaging of endosomes in live cells.

To confirm that the ECGreen endocytosis detection reagent (Dojindo) visualized endosomes in JAR-derived cells, NT1 cells transfected with a plasmid encoding an endosome marker protein, DsRed-Rab5 or DsRed-Rab7, were stained with the reagent and observed using a confocal fluorescence microscope (Fluoview FV3000). NT1 cells in eight-well chamber slides were inoculated with the DiD-labeled RuV at an MOI of 50. After incubation on ice for 1 h, the unbound viruses were removed by washing with RPMI 1640 twice, and then the cells were incubated in a medium containing the ECGreen endocytosis detection reagent at 37°C. After incubation for 2 h, live cells were observed using a confocal fluorescence microscope (Fluoview FV3000).

### Statistical analyses.

Each assay was performed in at least three independent experiments, and quantitative data are presented as the mean values of three experiments with error bars indicating the standard deviations. Statistical analyses and graphical representations were performed using GraphPad Prism version 9 software (GraphPad Software, San Diego, CA). The methods of statistical analyses are indicated in each figure legend.

### Data availability.

Raw data of deep sequencing during genome-wide CRISPR/Cas9 screenings have been deposited at DDBJ Sequence Read Archive under accession numbers DRA013199, DRA013200, and DRA013201.
