# Supplementary figures and images for: Membrane Sphingomyelin in Host Cells Is Essential for Nucleocapsid Penetration into the Cytoplasm after Hemifusion during Rubella Virus Entry
Source: mBio. 2022 Nov 8;13(6):e01698-22. doi: 10.1128/mbio.01698-22 (PMC9765692; doi:10.1128/mbio.01698-22)

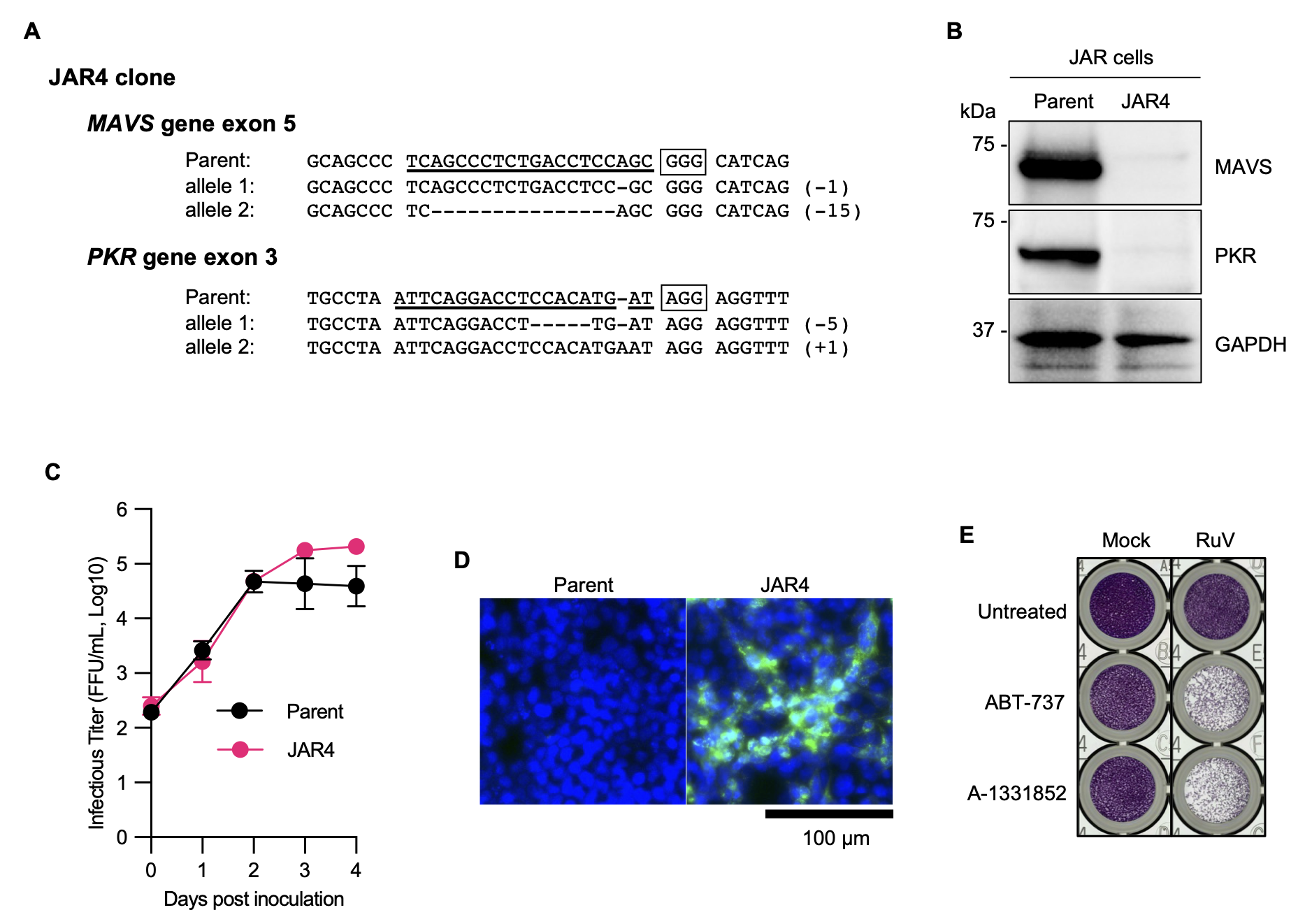

Supplement: FIG S1 [file mbio.01698-22-s0006.tif]

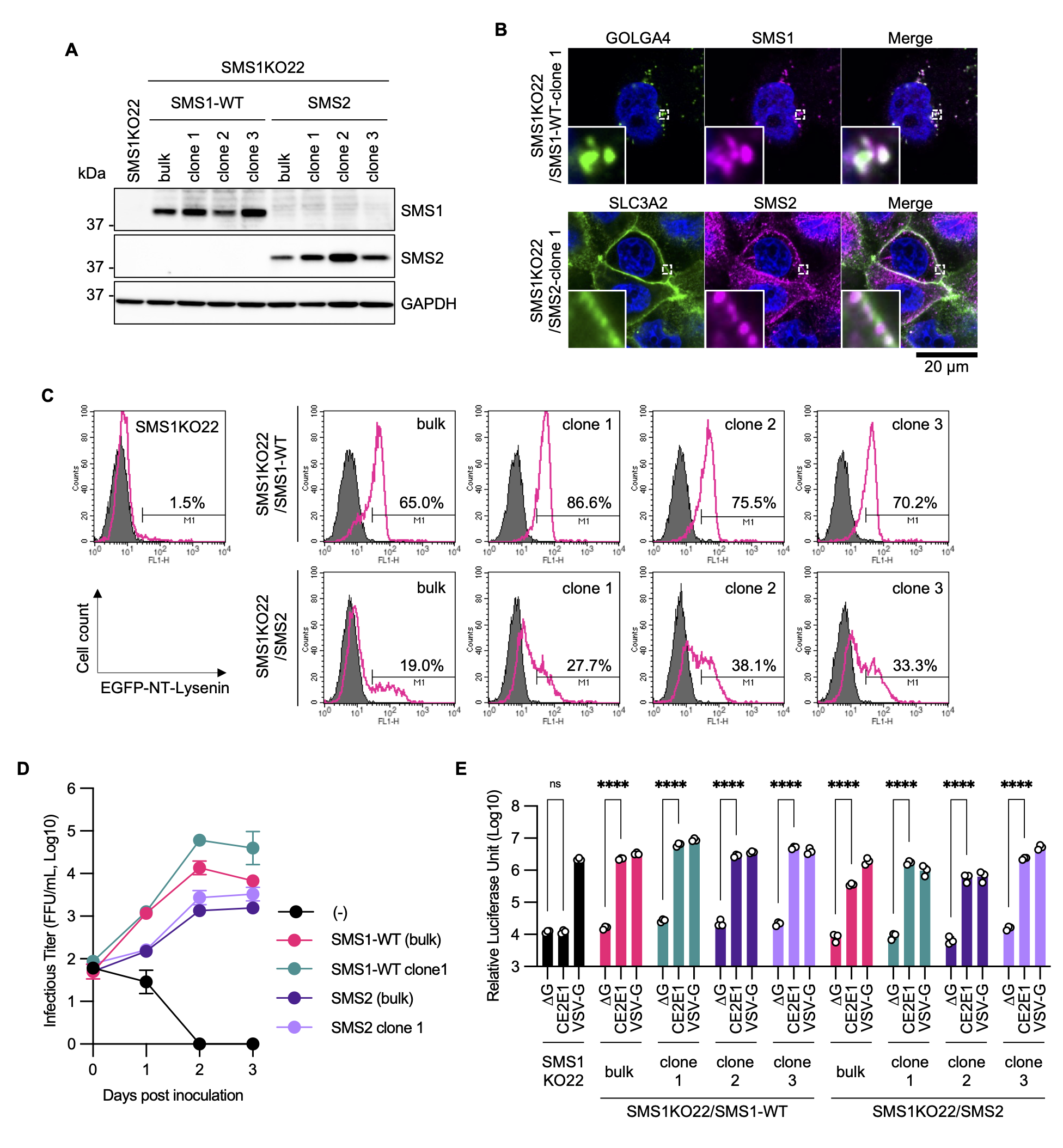

Supplement: FIG S2 [file mbio.01698-22-s0007.tif]

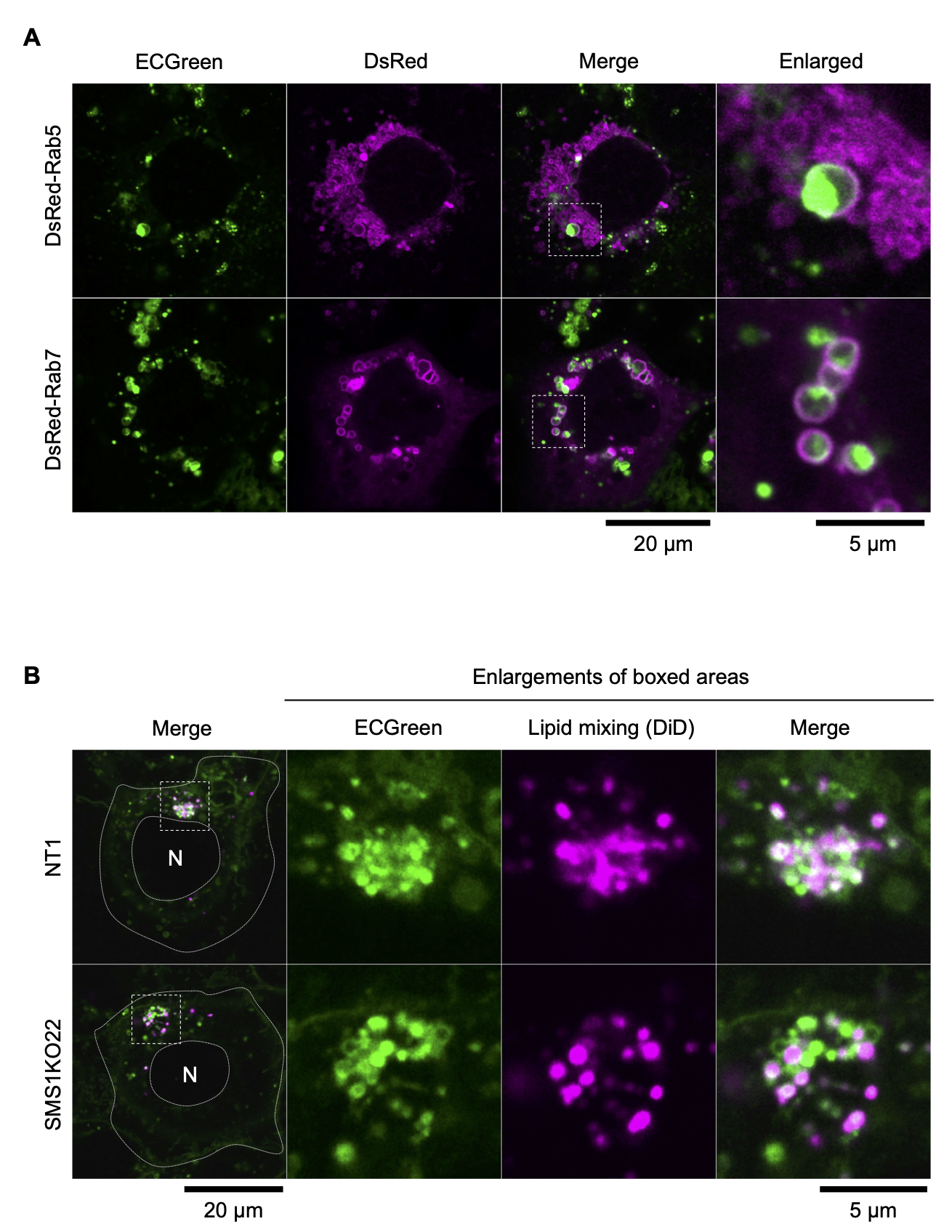

Supplement: FIG S3 [file mbio.01698-22-s0008.tif]

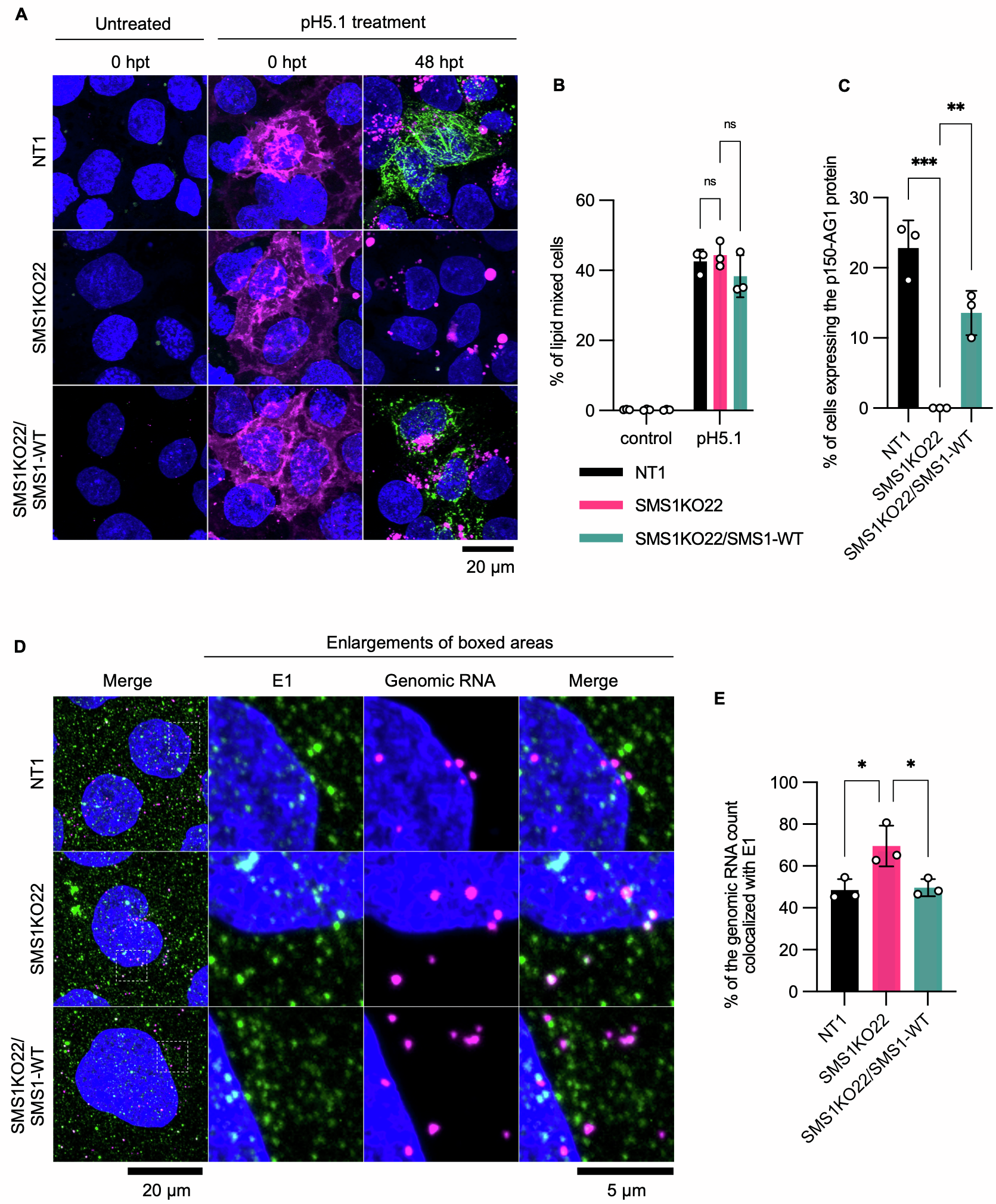

Supplement: FIG S4 [file mbio.01698-22-s0009.tif]

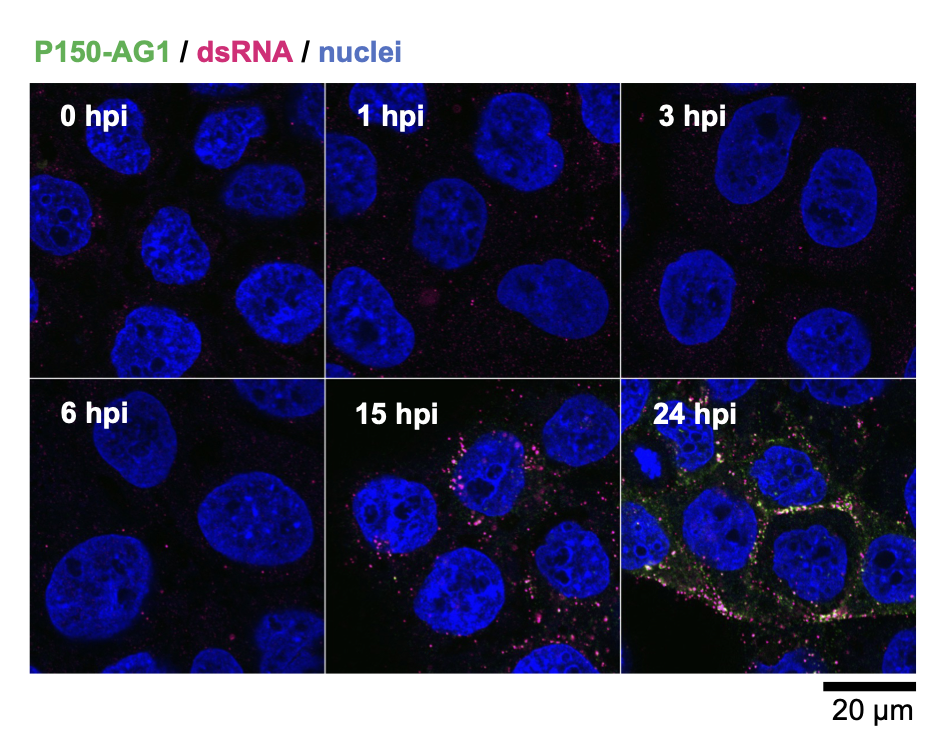

Supplement: FIG S5 [file mbio.01698-22-s0010.tif]
